# Neurocognitive modeling of latent memory processes reveals reorganization of hippocampal-cortical circuits underlying learning and efficient strategies

**DOI:** 10.1038/s42003-021-01872-1

**Published:** 2021-03-25

**Authors:** Kaustubh Supekar, Hyesang Chang, Percy K. Mistry, Teresa Iuculano, Vinod Menon

**Affiliations:** 1 Department of Psychiatry & Behavioral Sciences, Stanford University, Stanford, CA USA; 2 Developmental Psychology and Child Education Laboratory, University Paris Descartes, Paris, France; 3 Stanford Neuroscience Institute, Stanford University, Stanford, CA USA

**Keywords:** Cognitive neuroscience, Computational models, Learning and memory

## Abstract

Efficient memory-based problem-solving strategies are a cardinal feature of expertise across a wide range of cognitive domains in childhood. However, little is known about the neurocognitive mechanisms that underlie the acquisition of efficient memory-based problem-solving strategies. Here we develop, to the best of our knowledge, a novel neurocognitive process model of latent memory processes to investigate how cognitive training designed to improve children’s problem-solving skills alters brain network organization and leads to increased use and efficiency of memory retrieval-based strategies. We found that training increased both the use and efficiency of memory retrieval. Functional brain network analysis revealed training-induced changes in modular network organization, characterized by increase in network modules and reorganization of hippocampal-cortical circuits. Critically, training-related changes in modular network organization predicted performance gains, with emergent hippocampal, rather than parietal cortex, circuitry driving gains in efficiency of memory retrieval. Our findings elucidate a neurocognitive process model of brain network mechanisms that drive learning and gains in children’s efficient problem-solving strategies.

## Abstract (web summary)

Supekar, Chang, and Mistry et al. investigate behavioral and network-level changes in children after multi-week cognitive training for math problem-solving skills. After training, children show changes in modular network organization of hippocampal-cortical circuits that are correlated with gains in problem-solving accuracy and efficiency of memory retrieval. Taken together, these results suggest potential neurocognitive mechanisms underlying improved problem-solving abilities in children.

## Introduction

Early childhood is an important period for the acquisition of core competence in academically relevant cognitive domains^1–6^. Numerical problem-solving is one such domain of required core competence in modern societies: skills in this cognitive domain are important not only for professional success, health, and well-being later in life, but also for overall economic growth and prosperity in society at large^4,6,7^. Behavioral research has shown that the acquisition of numerical problem-solving skills in children is characterized by increased use of efficient memory retrieval-based strategies^4^. However, little is known about the neurocognitive mechanisms that underlie the acquisition of efficient memory retrieval-based problem-solving strategies. Here we investigate how cognitive training designed to improve children’s problem-solving skills alters brain network organization and leads to increased use and efficiency of memory retrieval-based problem-solving strategies. We developed, to the best of our knowledge, a novel neurocognitive process model to assess latent problem-solving strategies, and provide quantitively rigorous insights into how children develop specialized brain networks and acquire efficient problem-solving strategies, overcoming methodological limitations of previous studies. This knowledge has the potential to substantially advance our understanding of how brain network plasticity supports efficient learning in children and has implications for developing approaches for enhancing core competence in educational practice.

A key feature of the development of numerical problem solving is a shift from the use of inefficient counting to efficient memory-based strategies. For example, children learn arithmetic facts by progressing from frequent use of counting, through intermediate strategies, until they are eventually able to directly retrieve the answer from memory^8–11^. Typically, such changes are observed over the course of one or two years of schooling^12,13^, making it difficult to disentangle the contributions of learning from normative brain development. To address this, we used a short-term cognitive training program^14^ to investigate how learning dynamically alters the mix of strategies used, whether it improves the efficiency with which memory-based strategies are applied^11,15,16^, and how these changes are related to learning-related reorganization of the functional brain networks in children. Our cognitive training program, adapted from MathWise^17,18^, combined conceptual instruction with speeded retrieval of math facts and involved 22 lessons of increasing difficulty delivered over 8 weeks (see “Methods” for details). The training program centered on improving number knowledge and relations within and between numerical operations that facilitate the use of sophisticated counting procedures and memory-retrieval-based processes. A strategic practice component was included in the training program to promote the use of efficient problem-solving strategies, which then likely facilitates the formation and strengthening of long-term memory representations of math facts.

There is now growing evidence that learning involves changes in brain network organization^19–28^, including changes in modular architecture that guides efficient information flow in the brain to support adaptive behavior^20,29–31^. For instance, 6 weeks of motor sequence learning in adults leads to reduced functional integration between motor and visual modules and disengagement of cognitive control hubs in frontal and cingulate cortices, which suggests that acquisition of motor skills enhances specialization of sensorimotor subsystems^20^. Whether changes in modular network architecture also drive children’s acquisition of numerical problem-solving skills is not known. Considering that mathematical problem solving and learning involve distributed neural systems^32–36^, a more comprehensive understanding of how interactions between multiple brain networks dynamically change with skill acquisition and give rise to specialized functional modules^37,38^ is critically needed.

Building on recent advances in systems neuroscience models of numerical information processing^35^, the current study investigated functional reorganization of a network of brain regions important for numerical problem solving in children, including parietal, frontal, and ventral temporal-occipital regions^1,13,32,34,39–43^, as well as hippocampal and parahippocampal regions within the medial temporal lobe (MTL) learning and memory system, important for children’s math fact learning^13,39,40,44–46^. We used quantitative network connectivity analysis to investigate functional brain network reorganization in relation to learning gains and increases in the use of efficient problem-solving strategies. Task-relevant functional connectivity associated with numerical problem solving was examined, contrasting Addition (e.g., 3 + 4 = 7) and Control (e.g., 7 = 7) conditions to control for low-level perceptual processing and motor responses during numerical problem solving. Pairwise associations in connectivity strengths between all nodes were used to construct functional networks before and after training. We assessed training-induced changes in global modular brain network organization as well as changes in brain network organization at the regional level focusing on core functional circuits associated with mathematical learning in children: the MTL and the posterior parietal cortex (PPC). This approach allowed us to examine whether and how cognitive training leads to dissociable changes in the modular organization of MTL and PPC brain networks in children and identify functional circuits critically involved in the acquisition of memory-based strategies in numerical problem solving.

Recent functional brain imaging studies suggest a key role for the MTL, and particularly its hippocampal subdivision, in the longitudinal development of children’s numerical problem-solving skills^13,40,45,47^. The hippocampus is thought to be particularly important for early stages of learning and memory consolidation^48,49^ as well as binding of information together^49–55^, such as associating addends with sums in addition problems. More broadly, while the intraparietal sulcus (IPS) subdivision of the PPC has been specifically implicated in visuospatial representation and manipulation of numerical quantity in children and adults^1,42,43,56–61^, the hippocampus has been linked to learning and memory across different cognitive domains besides mathematics^62^. Consistent with this view, functional coupling between the hippocampus and frontoparietal brain regions has been linked to the use of memory-based problem-solving strategy and training-related arithmetic performance gains in children^13,39,45,46,56,63^. Notably, hippocampal connectivity has been shown to more strongly predict learning gains in numerical problem-solving skills than IPS connectivity^46^. Based on the theoretical framework that skill acquisition during development involves selective strengthening of functional circuits^37,38^ and the domain-general role of the MTL in learning, we hypothesized that learning-related functional reorganization would be driven by modular changes in MTL circuitry. The alternate hypothesis is that learning-related functional reorganization is selectively driven by modular changes in domain-specific IPS circuitry associated with numerical and visuospatial processing.

To assess children’s use of efficient problem-solving strategies, we developed, to the best of our knowledge, a novel neurocognitive process model of latent memory processes, which overcome several limitations of extant approaches. First, our computational modeling approach overcomes limitations inherent in assessments of problem-solving strategies using subjective verbal reports^64^, which can be biased by the nature of the instructions and queries, and by expectations of what constitutes desirable responses^65–69^. Second, this limitation is further confounded in fMRI studies by the inherent difficulties of assessing strategy use with verbal reports in the scanner^13,44–46,70,71^, thereby necessitating an additional problem-solving session outside the scanner in which children may not consistently use the same strategies. Computational modeling allowed us to more directly assess strategy use during fMRI task performance and provide clear insights into the procedural and chronometric dynamics of each strategy, without acquiring additional data outside the scanner. Third, circumventing issues associated with averaging all items in other approaches, our computational models address item-level heterogeneity and variability in task difficulty, allowing for more robust comparisons of pre- and post-training frequency and efficiency of strategy use. Lastly, compared to unidimensional overt behavioral measures such as accuracy and reaction time, multidimensional latent cognitive measures derived from computational models provide a set of fine-grained measures of individual differences in neurocognitive processes associated with problem solving^72,73^. Thus, dynamic cognitive modeling may have greater sensitivity to detect individual differences in numerical problem solving than overt behavioral measures, and thereby better characterize sources of individual differences in learning and brain plasticity.

Our cognitive process-based computational model (see “Methods” for details) probed children’s use and efficiency of problem-solving strategies in an unsupervised manner, for each individual on a trial-by-trial basis. Briefly, we modeled the problem-solving process as a mixture model of two latent strategies: memory retrieval and counting strategies—each as a distinct drift–diffusion process^74^. This model inferred which of these two strategies best explains performance (the joint distribution of accuracy and reaction time) on a trial-by-trial level, accounting for the variability in item difficulty levels across trials, for each individual. In this model, the selection of memory retrieval or counting strategy was characterized by the probability of shifting away from a primary, memory retrieval strategy, to an alternate counting strategy in a two-step strategy-selection process, dependent on both individual-level retrieval propensity and item-specific effects. The rate of evidence accumulation of the drift–diffusion process, based on a combination of individual level and item difficulty parameters unique to each strategy, determined the efficiency of a given strategy.

To further validate our model, we additionally implemented a second joint neurocognitive process model that jointly characterizes changes in problem-solving strategies and changes in brain circuits. This model, which integrates psychometric measurement, cognitive process modeling, and brain network analysis, was implemented within a hierarchical Bayesian inference framework^75^. By characterizing behavior using multiple dimensions of neurocognitive processes, our approach allowed us to more precisely measure training-induced changes in use and efficiency of memory-based strategies, and how changes in specific latent model parameters related to changes in brain network organization of functional circuits.

We hypothesized that cognitive training, focused on improving numerical problem-solving skills through strengthening retrieval of math facts, would lead to learning gains and changes in modular organization at the large-scale network level as well as at the regional level anchored in the hippocampus, rather than the IPS. We predicted that cognitive training would enhance hippocampal network modular segregation, as measured by lower levels of diversity coefficient^76^, and the degree of this functional reorganization would predict learning outcomes, as measured by gains in performance and latent measures of memory retrieval.

Our results show that cognitive training increases children’s use and efficiency of memory retrieval and improves their numerical problem-solving ability. Training-induced changes in functional brain network organization were characterized by increase in network modules as well as reorganization of hippocampal-cortical circuits associated with gains in efficiency of memory retrieval. Our findings elucidate a neurocognitive process model of brain network mechanisms that drive learning and gains in children’s efficient problem-solving strategies.

## Results

### Cognitive training improves performance on numerical problem solving

**Fig. 1:**
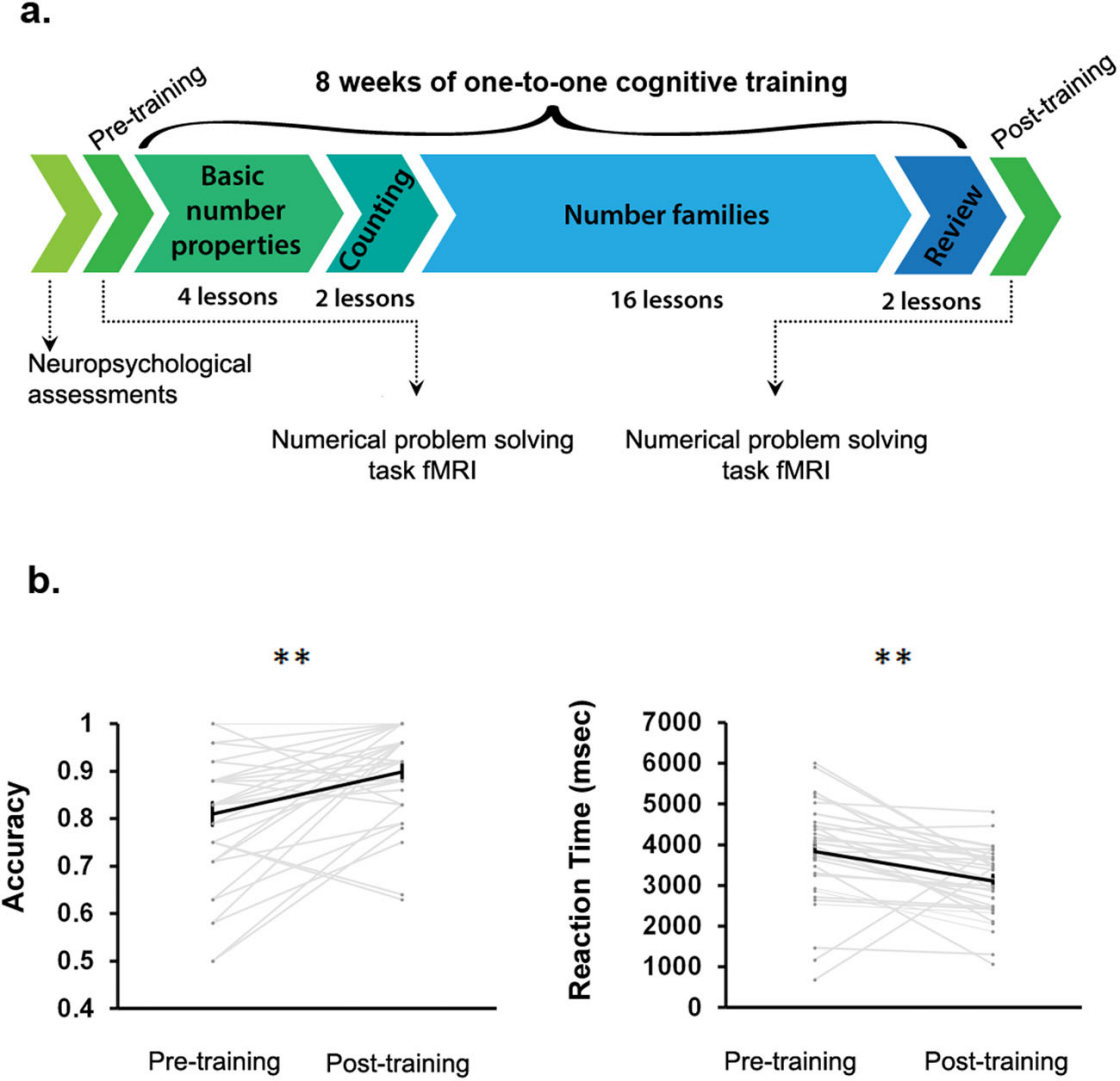
Cognitive training improves performance on numerical problem solving. **a** Overall study design. Before training, all children underwent an extensive battery of neuropsychological assessments for intelligence quotient (IQ) and academic achievement. Additionally, before training, each child underwent an fMRI scan session during which they completed Addition (verification of addition equations) and Control (verification of number identities) conditions in a numerical problem-solving task. Upon successful completion of the aforementioned sessions, children went through an intensive 8-week one-to-one training program focused on conceptual aspects of number knowledge and speeded practice on efficient problem-solving strategies and systematic learning of numerical problem solving delivered through 22 lessons of increasing difficulty. Training sessions occurred three times per week and were each 40–50 min in duration. After 8 weeks of training, all children underwent a second fMRI scan session. Adapted from Iuculano et al.^14^. **b** Numerical problem-solving skills (assessed by performance on Addition condition) improved significantly with training – performance gains were observed for both accuracy (*t*(34) = 3.98, *p* < 0.001, Cohen’s *d* = 0.75) and reaction time (*t*(34) = −3.68, *p* < 0.001, Cohen’s *d* = −0.70). *N* = 35 children. Error bar shows standard error of mean. **: *p* < 0.001. msec: millisecond.

To assess the efficacy of our cognitive training program (Fig. 1a), we first examined changes in accuracy and reaction time on a numerical problem-solving task involving verification of single-digit addition problems (e.g. 3 + 4 = 7). We found that children improved significantly with training—gains were observed for both accuracy (*t*(34) = 3.98, *p* < 0.001, Cohen’s *d* = 0.75) and reaction time (*t*(34) = −3.68, *p* < 0.001, Cohen’s *d* = −0.70) (Fig. 1b).

To assess the specificity of these gains, we then examined performance on a control task involving verification of number identity (e.g., 7 = 7). Here, changes in performance were not consistent across behavioral measures: while children showed decrease in reaction time (*t*(34) = −3.89, *p* < 0.001, Cohen’s *d* = −0.60), they did not show improvements in accuracy (*t*(34) = 0.91, *p* = 0.37, Cohen’s *d* = 0.20). Critically, changes in reaction time were not correlated between addition and control tasks (ρ = 0.15, *p* = 0.39). Additionally, changes in reaction time on the addition task remained significant even after controlling for changes in reaction time on the control task (*p* < 0.05). These results suggest that the training program was highly effective in that systematic and specific gains in numerical problem solving were seen in observable behavioral measures, including accuracy and reaction time, independent of changes associated with repeated exposure to the task or practice effects.

### Cognitive training increases the use and efficiency of memory retrieval-based problem-solving strategy

**Fig. 2:**
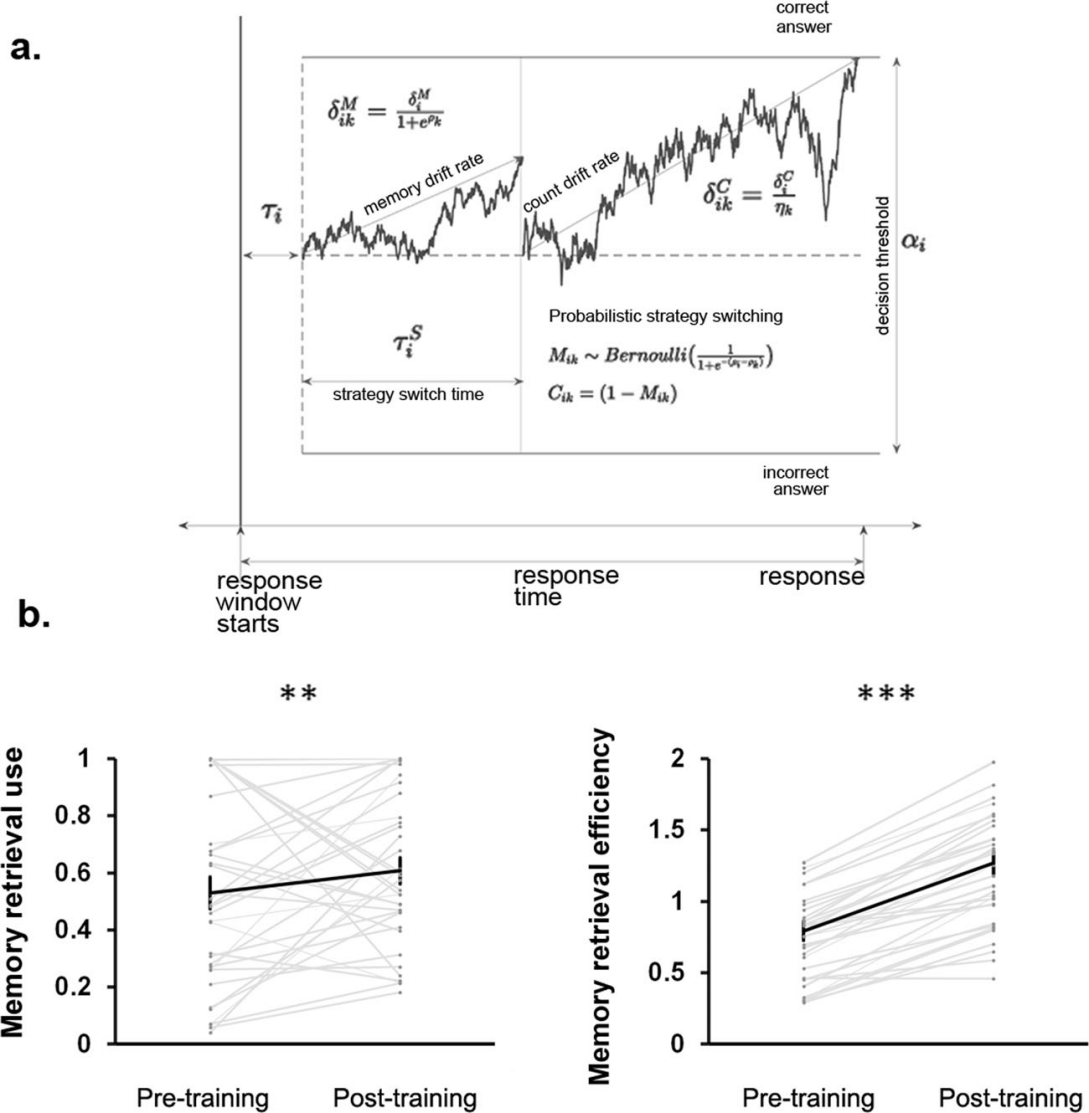
Cognitive training increases the use and efficiency of memory retrieval-based problem-solving strategy. **a** A dynamic latent cognitive model reveals multiple processes associated with problem-solving strategy use. The first drift–diffusion process shows an early terminated memory retrieval process governed by memory retrieval related drift parameters (e.g., memory drift rate), which has not reached the decision threshold when the strategy switching time is reached. At this point, a probabilistic decision is made to either continue with memory retrieval or shift to a counting strategy. This figure shows a shift to counting, with the drift–diffusion process in the second part being governed by counting related drift parameters (e.g., count drift rate; see “Methods” for details). **b** Children showed a significant increase in their use of memory retrieval strategy (*t*(34) = 3.20, *p* < 0.001, Cohen’s *d* = 0.26), after training. They also showed an increase in the efficiency of memory retrieval strategy (*t*(34) = 14.34, *p* < 0.0001, Cohen’s *d* = 1.07), as measured by memory retrieval drift rate, with training. *N* = 35 children. Error bar shows standard error of mean. ***: *p* < 0.0001, **: *p* < 0.001.

To examine the effect of the training program on latent processes associated with numerical problem solving, we performed a trial-by-trial analysis of the addition task using a two-component cognitive process model (Fig. 2a; Supplementary Figs. 1, 2; see also “Methods” for details). Children showed a significant increase in their propensity to use a memory retrieval strategy, a measure independent of difficulty of items presented, after training (*t*(34) = 5.36, *p* < 0.0001, Cohen’s *d* = 0.60). The actual use of memory retrieval strategy also significantly increased with training (*t*(34) = 3.20, *p* < 0.001, Cohen’s *d* = 0.26) (Fig. 2b). These changes in memory retrieval strategy use were accompanied by a significant increase in the efficiency of memory retrieval, as measured by memory retrieval drift rate, with training (*t*(34) = 14.34, *p* < 0.0001, Cohen’s *d* = 1.07) (Fig. 2b). These results suggest that the cognitive training led to an increase in memory retrieval strategy use, as well as increased efficiency and decisiveness in memory retrieval.

### Cognitive training related increases in the use and efficiency of memory retrieval-based problem-solving strategy relate to different aspects of observable behavioral measures

Training-related change in model-inferred memory retrieval efficiency was positively correlated with the change in accuracy (ρ = 0.42, *p* = 0.01), while the change in the use of memory retrieval strategy was negatively correlated with change in reaction time (ρ = −0.49, *p* = 0.01) (Supplementary Table 1). However, there was no significant relationship between change in memory retrieval efficiency and change in reaction time, nor between change in memory retrieval strategy use and change in accuracy (|ρ|s < 0.12, *p*s > 0.50) (Supplementary Table 1). These results demonstrate that latent cognitive measures of memory retrieval relate to different aspects of observable behavioral performance on the numerical problem-solving task.

### Cognitive training induces changes in modular brain network organization

**Fig. 3:**
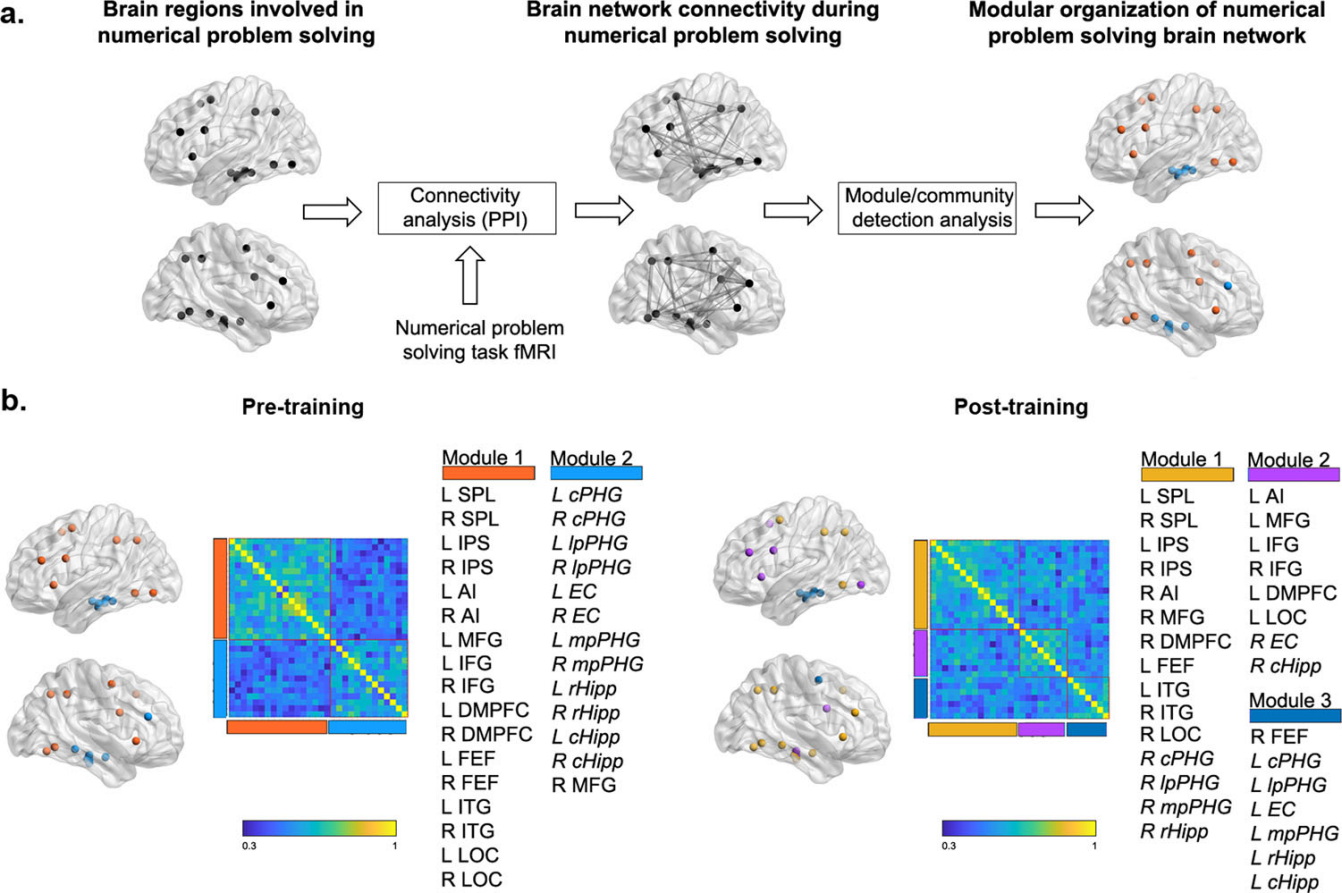
Cognitive training induces changes in modular brain network organization. **a** Overall analytical pipeline to compute and compare modularity of brain network organization before and after training. Meta-analysis was used to identify parietal, frontal, and ventral temporal-occipital regions consistently activated during numerical problem solving, in addition to hippocampus and parahippocampal gyrus subregions within the medial temporal lobe (MTL). We constructed brain network models using task-evoked measures of functional connectivity (estimated by Addition vs. Control condition) between these brain regions and used community detection analysis to investigate network organization before and after training. **b** Graph-based analysis revealed distinct patterns of modular network organization before and after cognitive training. Specifically, group-averaged task-related brain networks, as shown in connectivity matrices, were characterized by the presence of two modules prior to training and three modules after training. Before training, the first module (Module 1) consisted of parietal, frontal, and ventral temporal-occipital regions, while the second module (Module 2) comprised all MTL regions, including the hippocampus and parahippocampus bilaterally, and the right medial frontal gyrus (MFG). After training, individual functional subregions of the MTL formed a more complex three-module structure with parietal, frontal, and ventral temporal-occipital regions (Modules 1–3). *N* = 35 children. MTL regions are shown in italics. Color bar represents connectivity strength between pairs of nodes. SPL: superior parietal lobule; IPS: intraparietal sulcus; AI: anterior insula; IFG: inferior frontal gyrus; DMPFC: dorsomedial prefrontal cortex; FEF: frontal eye field; ITG: inferior temporal gyrus; LOC: lateral occipital cortex; cPHG: caudal parahippocampal gyrus; lpPHG: lateral posterior parahippocampal gyrus; EC: entorhinal cortex; mpPHG: middle posterior parahippocampal gyrus; rHipp: rostral hippocampus; cHipp: caudal hippocampus. L: left; R: right.

We next examined training-related changes in the modular organization of brain networks involved in numerical problem solving (Fig. 3a). Graph-based analysis revealed distinct patterns of modular network organization before and after training (Fig. 3b). Specifically, group-averaged task-related brain networks were characterized by the presence of two modules prior to training and three modules after training. Before training, the first module (Module 1) consisted of parietal, frontal, and ventral temporal-occipital regions, while the second module (Module 2) comprised the MTL memory system, primarily the hippocampus and the parahippocampal gyrus bilaterally in addition to the right MFG, whose connectivity with the MTL has been implicated in memory-based numerical problem solving^13,39^. Brain regions identified in Module 1 were among those most consistently activated during numerical problem solving across multiple studies, as determined by meta-analysis using Neurosynth^77^ (see “Methods”). After training, individual functional subregions of the MTL were no longer distinctly segregated and instead formed a more integrated three-module structure with parietal, frontal, and ventral temporal-occipital cortical regions involved in numerical problem solving.

Next, to determine whether training significantly altered modular network organization across individuals, we assessed the mutual information, a nonlinear measure of distance between modular structure before training and modular structure after training, in each participant. Distance between two modular structures was computed by subtracting mutual information between the two module affiliation vectors (ranging from 0 to 1) from 1. Thus, larger distance reflected greater training-induced functional brain reorganization. A two-tailed one-sample *t*-test contrasting the distance between pre- and post-training networks (*M* = 0.96*;* SD = 0.03) with the null hypothesis of no change in modular structure (distance = zero) revealed significant change in modular network organization with training (*p* < 0.001).

These results suggest that cognitive training changes overall modular network organization, characterized by reconfiguration of the MTL system into distinct patterns of MTL-cortical circuits in response to training in numerical problem solving.

### Cognitive training-induced modular brain network reorganization predicts performance gains

**Fig. 4:**
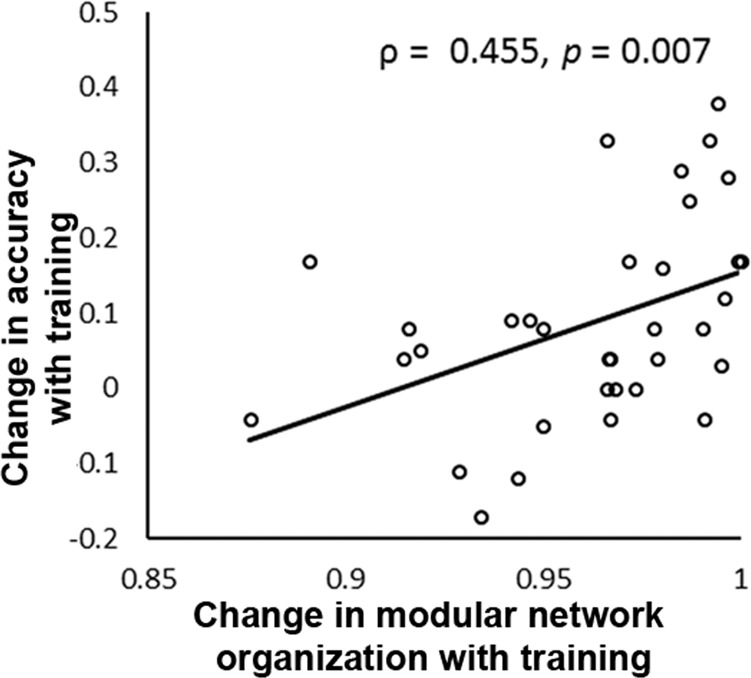
Cognitive training-induced modular brain network reorganization predicts performance gains with training. Children who showed greater training-induced changes in modular brain network reorganization, as indexed by a mutual-information-based distance metric, exhibited larger gains in accuracy on addition problems with training (ρ = 0.455, *p* = 0.007). *N* = 35 children.

We next investigated whether training-induced change in modular organization of brain network involved in numerical problem solving is associated with performance gains. Our analysis revealed a significant positive correlation (ρ = 0.46, *p* = 0.007) such that children who showed greater training-induced global functional brain reorganization, as indexed by distance between pre- and post-training networks, exhibited larger performance gains with training (Fig. 4). This result was specific to accuracy, as the result of additional analysis using reaction time was not significant (ρ = 0.18, *p* = 0.32). Post-hoc analysis revealed that changes in accuracy and reaction time were not significantly correlated (ρ = 0.18, *p* = 0.30). Furthermore, none of the behavioral measures included in the extensive battery of neuropsychological assessments conducted before training, including assessments of IQ and math and reading abilities, was associated with numerical problem-solving performance gain with training (|ρ|s < 0.31, *p*s > 0.06) (Supplementary Table 2). The correlation between modular network reorganization and accuracy gain was greater than any association between neuropsychological measures and accuracy gain (*p*s < 0.01). These results suggest that training-induced change in global modular brain network organization predicts performance gains in children.

### Cognitive training induces changes in node-level brain network organization

**Fig. 5:**
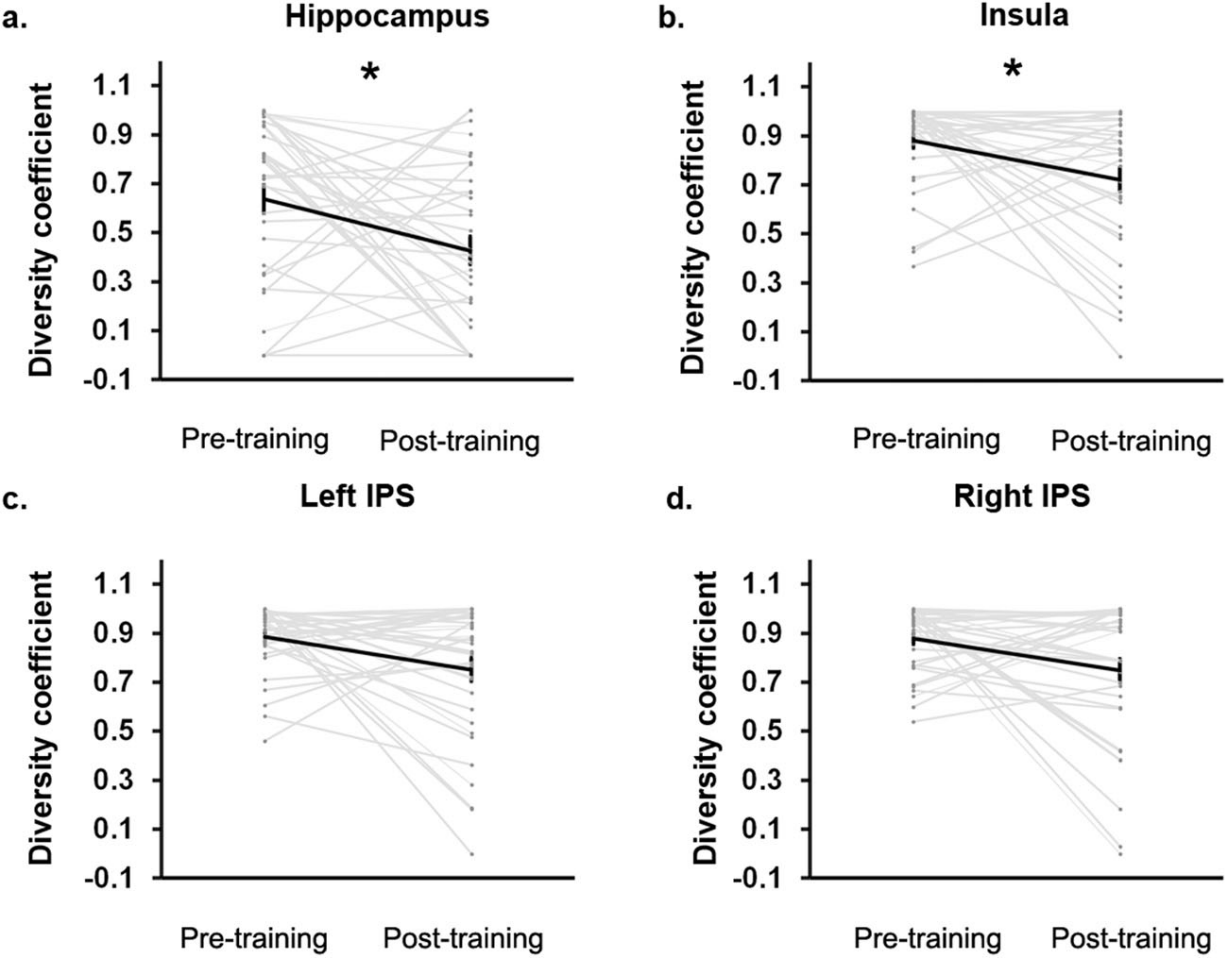
Cognitive training induces changes in network organization of the hippocampus and anterior insula. Diversity coefficient, a measure of how uniformly a brain region interacts with regions in other modules, of (**a**) the right rostral hippocampus and (**b**) the right anterior insula decreased with training (*p*s < 0.05, false discovery rate [FDR]-corrected). The diversity coefficient of (**c**, **d**) the bilateral intraparietal sulcus (IPS) —a brain area consistently implicated in numerical problem solving—did not change with training. *N* = 35 children. Error bar shows standard error of mean. *: *p* < 0.05.

In addition to global modularity changes, changes in brain network organization may occur at the regional level for each brain network node. To examine training-related changes in the regional organization of brain networks involved in problem solving, we used the modular structure derived above to compute a region-wise diversity coefficient—a measure of how uniformly a brain region interacts with regions in other modules. A high value for the diversity coefficient indicates that interactions are more evenly distributed across many modules, while a low value indicates interactions with fewer modules, or increased modular segregation^76^. We found that the diversity coefficient of the right rostral hippocampus and the right anterior insula decreased with training (*p*s < 0.05, FDR corrected) (Fig. 5a, b). No other regions including the bilateral IPS showed significant training-related changes in the diversity coefficient (Fig. 5c, d; Supplementary Fig. 3). As left and right IPS nodes show similar patterns of results, we subsequently report diversity coefficient of bilateral IPS region, combining the left and right IPS nodes. An exploratory analysis of variance (ANOVA) yielded no significant Region (right rostral hippocampus, bilateral IPS) by Time interaction (pre-training, post-training) (*F*(2, 34) = 0.62, *p* = 0.61). As numerical problem solving involves distributed functional circuits, it is possible that weak (non-significant) modular changes in domain-specific IPS circuitry occur along with significant changes in domain-general hippocampal circuitry. Nonetheless, our key findings suggest that the functional interactions of the right rostral hippocampus and the right anterior insula become less diverse with training, demonstrating functional specialization of these regions associated with acquisition of numerical problem-solving skills.

### Cognitive training-induced right rostral hippocampal network reorganization predicts performance gains

**Fig. 6:**
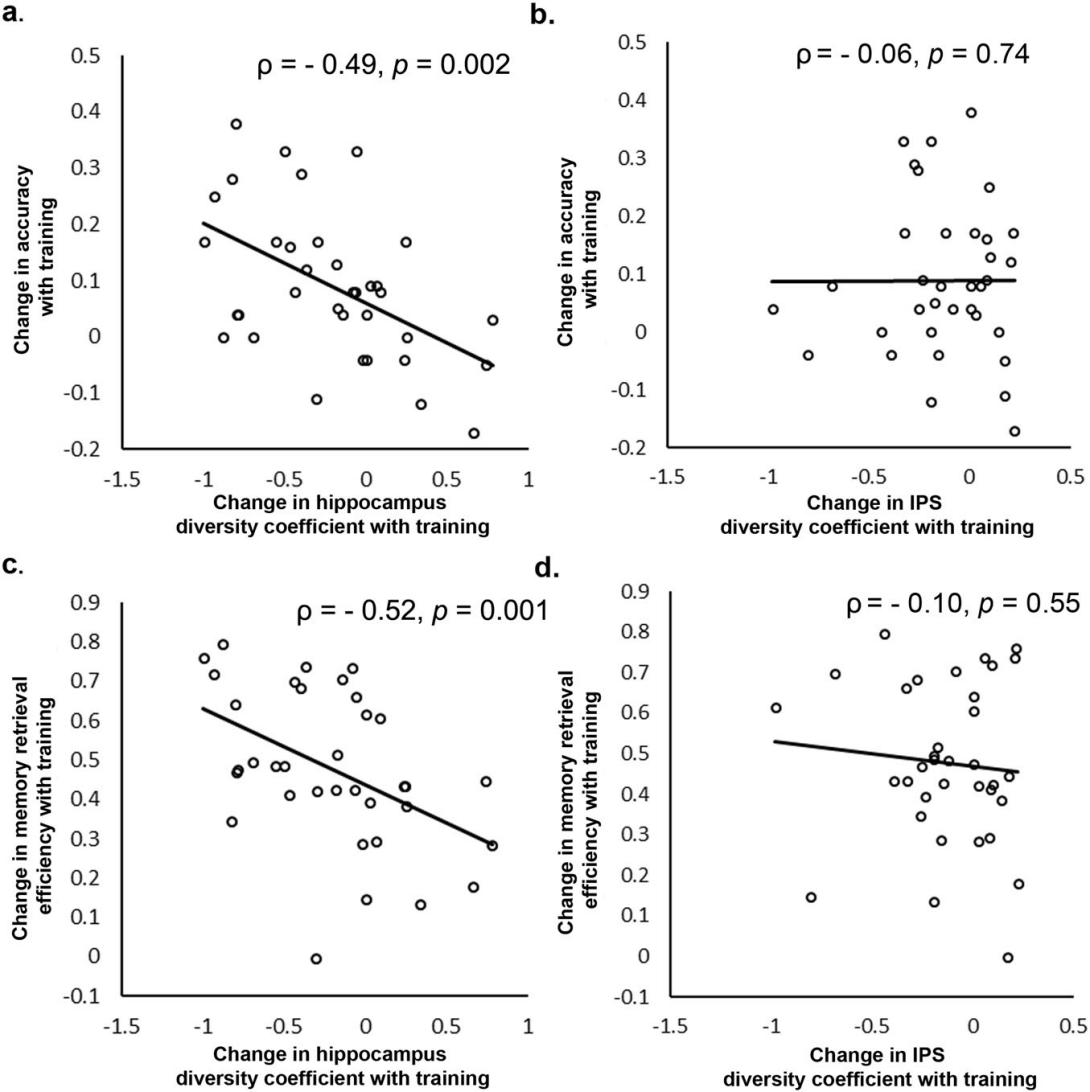
Cognitive training-induced network reorganization of the hippocampus predicts learning and memory retrieval efficiency gains. **a** Training-related change in diversity coefficient of the right rostral hippocampus was significantly negatively correlated with performance gains (ρ = −0.49, *p* = 0.002), such that children who showed greater decreases in diversity coefficient, exhibited larger gains in performance with training. **b** Changes in the intraparietal sulcus (IPS) diversity coefficient with training were not correlated with performance gains with training. **c** Training-related change in diversity coefficient of the right rostral hippocampus was significantly negatively correlated with gains in efficiency of memory retrieval (ρ = −0.52, *p* = 0.001), such that children who showed greater decreases in diversity coefficient, exhibited larger gains in efficiency of memory retrieval with training. **d** Changes in IPS diversity coefficient with training were not correlated with gains in memory retrieval efficiency with training. *N* = 35 children.

We next assessed the relation between training-induced changes in the right rostral hippocampus and the right anterior insula network organization and change in accuracy on the numerical problem-solving task. Training-induced change in the diversity coefficient of the right rostral hippocampus was significantly negatively correlated with change in accuracy (ρ = −0.49, *p* = 0.002), such that children who showed greater decreases in the diversity coefficient, exhibited larger performance gains with training (Fig. 6a). This finding was specific to accuracy, as the result of additional analysis using reaction time was not significant (ρ = −0.19, *p* = 0.27). Furthermore, training-induced change in the diversity coefficient of the right anterior insula was not significantly correlated with performance gain (ρ = 0.15, *p* = 0.38). Change in the IPS diversity coefficient with training was also not correlated with performance gain (ρ = −0.06, *p* = 0.74) (Fig. 6b). These results suggest that training-induced changes in regional organization of the right rostral hippocampus specifically relate to training-induced performance gains in children.

### Cognitive training-induced right rostral hippocampal network reorganization predicts memory retrieval efficiency gain

We next assessed the relation between training-induced change in the right rostral hippocampus, the right anterior insula, and the IPS network organization and changes in memory retrieval strategy use and efficiency.

Training-induced change in the diversity coefficient of the right rostral hippocampus was significantly negatively correlated with change in memory retrieval efficiency (ρ = −0.52, *p* = 0.0014), such that children who showed greater decreases in the diversity coefficient, exhibited larger gains in memory retrieval efficiency with training (Fig. 6c). This result was specific to memory retrieval efficiency, as change in memory retrieval strategy use was not significantly associated with change in hippocampal network reorganization (ρ = 0.13, *p* = 0.44). Furthermore, changes in the right anterior insula and the bilateral IPS diversity coefficient with training were not correlated with gain in memory retrieval efficiency (|ρ|s < 0.10, *p*s > 0.10) (Fig. 6d). The correlation between change in memory retrieval efficiency and change in diversity coefficient was significantly different between the right rostral hippocampus and IPS nodes (*p* = 0.02).

The relationship between change in the diversity coefficient of the right rostral hippocampus and memory retrieval efficiency gain was validated by a second joint neurocognitive process model, where the model parameters were inferred by characterizing change in the diversity coefficient as a linear combination of change in the latent drift–diffusion model parameters. Here, we computed Bayes factors (BFs) using the Savage–Dickey computation method^78^ to test the relationship between latent parameter changes in memory retrieval strategy use and efficiency and change in the diversity coefficient of the right rostral hippocampus. BF values greater than 3 in favor of H_1_ provide moderate evidence for H_1_, those between 1/3 and 3 provide insufficient evidence, and those below 1/3 provide evidence of absence (i.e., BF > 3 in favor of the null hypothesis)^79^. The model revealed that training-induced change in memory retrieval efficiency was significantly associated with change in the diversity coefficient of the rostral hippocampus (β = −0.79, BF = 5.6). There was insufficient evidence in favor or against an association between training-induced change in the propensity to use memory retrieval or the actual use of memory retrieval and change in the diversity coefficient of the rostral hippocampus (1/3 < BF < 3). Training-induced change in decision threshold or switching time was not associated with change in the diversity coefficient of the rostral hippocampus (BFs > 3 in favor of the null hypothesis).

Finally, we assessed whether training-induced global change in the modular network organization, as assessed using mutual information-based distance metrics, relate to change in memory retrieval strategy use or efficiency. Global network change was not correlated with change in memory retrieval efficiency (ρ = 0.07, *p* = 0.71) or use (ρ = −0.20, *p* = 0.25), which suggests that training-induced gain in memory retrieval efficiency is specifically associated with right rostral hippocampal network reorganization.

Taken together, these results provide evidence that training-induced changes in regional organization of the right rostral hippocampus drive training-induced memory retrieval efficiency gains in children.

## Discussion

We used a neurocognitive process model to investigate whether cognitive training designed to improve children’s problem-solving skills alters the modular organization of hippocampal-cortical circuits and drives the use of efficient memory-based problem-solving strategies. We found that 8 weeks of cognitive training not only improved performance as indexed by observed behavioral measures, but also increased latent model-derived measures of memory retrieval strategy use and efficiency. Furthermore, cognitive training led to reduced diversity of intermodular functional connections of the right rostral hippocampus region of the MTL memory system and the right anterior insula node of the salience network. Notably, training-related changes in diversity of intermodular functional connections of the right rostral hippocampus predicted gains in efficiency of memory-based strategies. These effects were specific to the hippocampus as the intermodular functional connections of the IPS—a brain area consistently implicated in numerical problem solving—neither changed with cognitive training nor were they associated with individual learning gains.

Our findings demonstrate that behaviorally-relevant functional reorganization of the hippocampal-memory network supports distinct aspects of cognitive skill acquisition in children^13,40,45,47^. By examining learning-dependent plasticity of a distributed functional network involving the MTL memory system and multiple cortical regions consistently implicated in numerical problem solving, the current work provides key evidence that cognitive skill acquisition reorganizes specialized brain circuits in school-age children. These findings provide important insights into neurocognitive mechanisms underlying individual differences in children’s learning and have broad implications for educational practice and interventions for enhancing core competence in academically relevant domains.

The first key finding of our study is that our short-term cognitive training program was effective with systematic gains that were seen in observable behavioral measures as well as latent model-derived measures of changes in the rate of memory retrieval-based strategy use and efficiency. Consistent with previous developmental^11,44–46,63^ and classroom-based^17^ studies, short-term training improved (observed) performance on numerical problem solving. Crucially, our modeling of latent problem-solving strategies revealed that 8 weeks of cognitive training decreased the use of counting and increased the use and efficiency of memory-based retrieval strategy. These findings are consistent with the ‘overlapping waves’ theory^80^ which posits that children’s learning in arithmetic problem solving is characterized by changes in distributions of problem-solving strategies, rather than broad and abrupt shifts between processing stages^11^. Notably, our latent cognitive measures of memory retrieval strategy use and efficiency captured different aspects of observable behavioral measures: increase in memory retrieval strategy use was associated with faster reaction time and greater memory retrieval efficiency correlated with improved accuracy on numerical problem solving. Our findings suggest that acquisition of numerical problem-solving skills is characterized by greater use of memory-based retrieval strategies as well as greater efficiency in memory-based strategies, accompanied by faster problem solving and higher accuracy, respectively. More broadly, our computational modeling approach provides a quantitative template for investigating latent cognitive processes beyond observable behavioral measures in other domains (see Supplementary Discussion for more details).

The second key finding of our study is that cognitive training markedly altered the network organization of brain regions involved in numerical problem solving. Functional brain changes were characterized by changes in the modular brain network organization with training. To date, cognitive training studies have mainly focused on activation and connectivity of brain regions at single time points^23,28^, rather than plasticity of brain network configuration across time in response to training. Furthermore, most previous studies have been carried out in adults, and functional brain network reorganization in response to training has not been examined in children. Our study addresses these gaps and provides evidence for intervention-related plasticity in the modular organization of brain networks during problem solving in children. Specifically, we found that modular network organization changed with training. Notably, all subregions of the MTL, which initially belonged to a single, segregated module prior to training, were subsequently reorganized into three modules each with a distinct pattern of MTL-cortical circuits (Fig. 3b).

Our analysis further identified the right rostral hippocampus as the node that drives modular reorganization in response to cognitive training. Specifically, we found that the proportion of intermodular connections of the right rostral hippocampus was significantly reduced after training (Fig. 5a). A key aspect of this change was that, post-training, the rostral hippocampus was no longer part of the segregated MTL module but was instead now integrated into IPS, ITG, and LOC brain regions consistently implicated in numerical processing^1,35,41–43,56,57,59,61^ (Fig. 3b). In parallel, this reconfiguration was also accompanied by segregation of the rostral hippocampus-containing module from the two other modules, as indicated not only by the formation of a new functional module but also by a decrease in its diverse interactions as noted above. In other words, the right rostral hippocampus not only switched module allegiance from the MTL to a cortical IPS-ITG-LOC numerical processing module, but its interactions with other modules decreased at the same time.

By incorporating multiple MTL subdivisions in our large-scale brain network model, our results also help elucidate the differential role of the right rostral hippocampus in response to cognitive intervention. Previous memory studies have suggested that the rostral hippocampus is crucial for pattern completion, whereas the caudal hippocampus is crucial for pattern separation^81^. Thus, the rostral hippocampus subdivision of the MTL may contribute to the integration of memories by altered modular configuration and enhanced interactions with distributed brain regions implicated in numerical processing. Taken together, our findings suggest that network plasticity and reorganization of rostral hippocampus circuits underpins learning of academically relevant skills, in line with the integrative coding mechanisms proposed for this brain region^82–84^.

The third important finding of our study is that the training-induced brain network reconfiguration, both at the large-scale and regional levels, was associated with behavioral changes. Specifically, children who showed greater training-induced large-scale functional brain network reorganization, as measured by mutual information-based distance metrics, exhibited larger performance gains with training. Additionally, functional brain network reorganization better predicted performance gains with training than neuropsychological assessments acquired before training, including domain-general (IQ and reading) and domain-specific (numerical operations and math reasoning) measures. Furthermore, building on evidence showing brain network changes associated with the right rostral hippocampus, we found that children who showed greater training-induced functional brain reorganization of the right rostral hippocampus also exhibited larger gains in accuracy. Taken together, these results suggest that the degree of training-induced changes in functional network organization, both at the large-scale level and at the regional level localized to the right rostral hippocampus, are associated with individual differences in training-induced performance gains.

The fourth key finding of our study relates to changes in strategy use, as assessed with computational modeling that allowed us to quantify network mechanisms underlying greater use of memory-based strategies. Again, building on our finding of brain network changes associated with the right rostral hippocampus, we found that children who showed greater training-induced functional brain reorganization of the right rostral hippocampus also exhibited larger gains in memory retrieval efficiency. Notably, unlike overall changes in performance gains, as noted above, changes in memory retrieval efficiency were not significantly associated with large-scale network changes as assessed using mutual information-based distance metrics. Rather, gains in memory retrieval efficiency were specifically associated with the right rostral hippocampus region that showed significant reorganization with training. This dissociation further clarifies the specific role of emergent rostral hippocampus circuitry in driving the use of more efficient memory-based problem-solving strategies. The specificity of the association between changes in memory retrieval efficiency, rather than overall performance gains, and right rostral hippocampal network reorganization is further highlighted by our control analysis (Supplementary Fig. 4), which shows that the strategy process dissociation model provides a significantly superior characterization of the change in right rostral hippocampal brain network organization, compared to a control model that applies canonical drift–diffusion processes without inferring latent strategies.

Taken together, the current study demonstrates that a short-term cognitive training, designed to improve children’s problem solving, alters functional brain network organization and leads to increased use and efficiency of memory retrieval-based strategies in children.

Future studies will need to address two limitations. First, it is possible that some of the changes in brain and behavior observed in this sample may have arisen from practice effects or repeated testing, which is also known to facilitate learning^85–87^. Follow-up experiments with a well-matched active control will be needed to better understand training-specific learning and functional brain reorganization. Second, our neurocognitive process models of numerical problem-solving strategies were based on single-digit addition problems. Further studies are needed to validate latent cognitive processes involved in solving more complex numerical problems and training-related transfer to new problems, including the possibility of use of more than two strategies, beyond retrieval and counting. Additionally, longitudinal follow-up studies are needed to determine the long-term stability of the observed training-induced behavioral, cognitive, and neural changes. Finally, future work should investigate whether network-analytic quantitative measures of hippocampal-cortical network organization could serve as a brain-based biomarker for tailoring various cognitive training interventions.

In conclusion, the present work provides a comprehensive characterization of brain network mechanisms that drive academically relevant learning in children. Our quantitative network analysis combined with a computational modeling approach substantially improves our understanding of brain network mechanisms underlying the increased use of efficient memory-based problem-solving strategies. The current work presents a neurocognitive process model of latent memory processes that underlie individual differences in learning in response to cognitive training. More generally, our findings provide, to the best of our knowledge, novel evidence for theoretical models that posit that the emergence of brain network modules supports the development of specialized cognitive functions.

## Methods

### Experimental design

The current study examined the neurocognitive mechanisms that underlie the acquisition of efficient memory-based strategies, following a short-term cognitive training. Participant characteristics, study design, and procedures are described in the respective sub-sections below.

### Participants

Participants were recruited from multiple school districts in the San Francisco Bay Area. Participants had no history of psychiatric illness, neurological disorders, or reading disabilities. Informed consent was obtained from the legal guardian of each child and all study protocols were approved by the Stanford University Institutional Review Board. Thirty-five children in grade 3 (age: *M* = 8.58, SD = 0.58, 20 females) participated in the current study (Supplementary Table 3).

### Overall study design

Figure 1a illustrates our study design. Demographic, neuropsychological, cognitive, and brain imaging measures were acquired from each participant prior to training. After successful completion of the MRI scanning session, children started an 8-week math training program. Training sessions occurred three times per week and were each approximately 40–50 min in duration. Response to training was examined using arithmetic verification and production tasks which assessed accuracy, reaction time, and retrieval strategy use before and after training.

### Neuropsychological assessments

All participants underwent a comprehensive battery of standardized neuropsychological assessments including the Wechsler Abbreviated Scale of Intelligence (WASI, 1st edition)^88^ and the Wechsler Individual Achievement Test (WIAT-II, 2nd edition)^89^ (Supplementary Table 3). IQ was determined using the WASI; academic achievement in reading and mathematics was assessed using the WIAT. These standardized measures were acquired prior to training and were not repeated because of the statute of limitations regarding their repeated use within a year.

### Training sessions

Children took part in an 8-week cognitive training program adapted from MathWise^17,18^. The training program combined conceptual instruction with speeded retrieval of math facts^14^. Similar to MathWise, the training involved a total of 15–20 h of training, but it was condensed to 8/9 weeks with longer lessons in order to equate overall time on training^14^. The training consisted of 22 lessons of increasing difficulty^14^. Lessons 1 through 4 reviewed adding and subtracting 0, 1, and 2, as well as low ties (from 1+1 to 6+6 and corresponding subtraction facts, e.g., 12–6)^14^. These lessons also taught the commutative property of addition (i.e., changing the order of the operands does not change the sum), as well as the additive identity property of zero (i.e., adding zero does not change the number’s value), and introduced the children to math manipulatives (i.e., a number line and blocks in a circle)^14^. Lessons 5 and 6 taught the min strategy for addition (i.e., start with the larger number and count up with the smaller number)^17,18^ and the missing addend strategy for subtraction (i.e., start with the smaller number and count up to the larger number)^14^. During lesson 7–22, children practiced with progressively more difficult problems^14^. They started out with all the addition problems that summed to 5, and the corresponding subtraction problems^14^. By the end of training, they learned addition problems that summed to 18, and their corresponding subtraction problems^14^. All lessons followed the same structure: (1) warm-up flashcards to review previously trained math problems; (2) number knowledge review, including the use of manipulatives and the counting strategies; (3) a lesson worksheet to introduce the new math problems; (4) a math game, (5) computerized flashcards combining the current and previous lessons’ material, (6) a physical flashcard game, and (7) a review worksheet of that day’s problem set^14^. Since scanning occurred only on weekends, children who completed lesson 22 early in the week took part in 1 or 2 additional review sessions^14^. Training was administered by well-trained research assistants, under the guidance of post-doctoral fellows to ensure fidelity of training implementation. To maximize compliance, participants were provided as many breaks as needed and were given positive feedback and incentives for completing training activities (stickers and small prizes)^14^.

### Training outcome measures

Response to training was examined using accuracy and reaction time (assessed in the fMRI scanner) and strategy use (assessed outside the scanner) on single-digit addition problems before and after training. While children were trained on both addition and subtraction problems as part of an established math training protocol^14^, our main outcome measures focused on addition problem-solving skills to probe memory-retrieval-based problem solving strategy use, which is more often observed in addition than subtraction problem solving^90–92^.

### Statistics and reproducibility

The current study used the following analytical approaches: (i) neurocognitive process model of latent memory processes to examine training-induced changes in the use and efficiency of memory-based problem-solving strategies and (ii) quantitative functional brain network analysis to investigate training-induced changes in modular organization of functional brain circuits. Two-tailed paired t-tests and Wilcoxon signed rank paired tests were performed for comparisons between pre- and post-training for behavioral and brain measures respectively, and Spearman correlation was used for analysis on brain-behavior relation, unless otherwise specified. Effect sizes, Cohen’s *d*, and Spearman’s rho, were estimated in Matlab. All statistical analysis is based on the sample of 35 children. Details on computational modeling and functional MRI network analysis are described in their respective sub-sections below.

### Computational modeling

#### Overview

We used computational modeling to assess the use and efficiency of problem-solving strategies. We modeled the problem-solving process as a mixture model of two latent strategies: memory retrieval and counting strategies (Fig. 2a). Our computational modeling allowed us to measure different strategy-specific process components, and their relation to changes in observable behavior and brain network organization. Our model is characterized by a process dissociation structure that imposes theoretically derived structural constraints on the trial-by-trial inference about which latent strategy is being used. The inference about which strategy was used on each trial is made by comparing the likelihood of behavioral responses (correct or incorrect choice and reaction time) under the parameters inferred for each individual strategy (Fig. 2a; Supplementary Figs. 1, 2).

The process dissociation model infers which of these two strategies best explains performance (the joint distribution of accuracy and reaction time) on a trial-by-trial level, accounting for the variability in item difficulty levels across trials, for each individual, given the observed data. Specifically, both the memory retrieval and counting strategies are modeled as distinct drift–diffusion processes^74^, with different start (non-decision) times, and a common decision threshold that measures the degree of evidence required to decide. Each drift–diffusion process represents a distinct cognitive problem-solving strategy (memory retrieval or counting). On each trial, an individual is assumed to follow a two-step strategy-selection process, with memory retrieval as the primary (default) strategy and a possible subsequent switch to an alternate counting strategy. This probabilistic strategy selection is characterized by adapting sequential item-response or SRM-MC models^93^, such that the probability of shifting away from a primary-memory-based strategy is dependent on both an individual-level propensity but also an item-specific effect that is common across all individuals.

The two-step strategy selection process is governed by a latent measure of executive function that characterizes the time taken for internal strategy switching. The rate of evidence accumulation of the drift–diffusion process is a measure of the efficiency of that strategy. This drift rate for memory retrieval is based on a combination of individual-level latent measure of memory retrieval efficiency as well as a latent item difficulty parameter which is estimated from the data across participants using an adapted form of item-response theory. For counting, the evidence accumulation process is inversely related to the number of counts required for each item, modulated by an individual level counting efficiency measure.

As an additional measure, we also implemented a second neurocognitive process model that jointly characterizes changes in problem-solving strategies and changes in brain circuits to provide precise measurements about how training-induced changes in different latent model parameters are linked to changes in brain modularity. This model, which integrates psychometric measurement, cognitive process modeling, and brain network analysis, was implemented within a hierarchical Bayesian inference framework (Supplementary Figs. 1, 2) using JAGS version 4.3.0^75^. The key imperative is that while typical approaches reduce a sequence of behavior into one or two dimensions of performance, our approach allows us to characterize behavior using multiple dimensions of individual differences, measure training-induced changes in each dimension, and relate changes in brain modularity to specific dimensions rather than overall performance gains.

#### Model and implementation

Multidimensional latent measures of individual differences were examined by computational modeling of behavioral responses during the arithmetic verification task. Specifically, the behavioral responses—choice accuracy and reaction time—were modeled as a drift–diffusion process (DDM), with the model implemented as a Wiener distribution^94,95^ with four parameters, the decision threshold, drift rate, bias, and non-decision time. Children use multiple problem-solving strategies (memory retrieval and counting), and each strategy is characterized by its own set of parameters that reflect mechanistic and chronometric assumptions about the strategies. Behavior on any trial (i.e., for any specific problem) is thus characterized as being a result of a probabilistic selection of one of these strategies and modeled as a mixture model of memory retrieval and counting strategies.

As noted above, the model assumes a sequential two-step process, with an initial attempt for memory retrieval followed by the possible application of a counting strategy. This implies a switching point at which an individual might give up on memory retrieval and switch to an alternate strategy or decide to continue with memory retrieval. We denote this as the strategy switching time. The probability of an individual *i* selecting a memory retrieval strategy for an item *k* depends on the individuals’ propensity towards memory retrieval *ρ*_*i*_ as well as how amenable the item is to be retrieved from memory *ρ*_*k*_. The *ρ*_*k*_ parameter can be interpreted as the degree of difficulty of memory retrieval for item *k* and is measured at the group level. Importantly, this dissociates the probability of memory retrieval into individual and item-level effects:

1 $$\begin{array}{*{20}{c}} {p\left( {{\mathrm{memory}}\;{\mathrm{retrieval}}} \right) = \frac{1}{{1 + {\rm{e}}^{ - \left( {\rho _i - \rho _k} \right)}}}} \end{array}$$

2 $$\begin{array}{*{20}{c}} {p\left( {{\mathrm{counting}}} \right) = \frac{{{\rm{e}}^{ - \left( {\rho _i - \rho _k} \right)}}}{{1 + {\rm{e}}^{ - \left( {\rho _i - \rho _k} \right)}}}} \end{array}$$

The memory retrieval process is characterized as a drift–diffusion process and the efficiency of memory retrieval is characterized by a memory retrieval drift rate parameter $\left( {\delta _{ik}^M} \right)$, with higher values characterizing faster and more accurate memory retrieval. The efficiency of memory retrieval is characterized as being dependent on an individuals’ memory retrieval efficiency $\left( {\delta _i^M} \right)$ and the nature of the specific item, specifically, the item *k* level difficulty of memory retrieval:

3 $$\begin{array}{*{20}{c}} {\delta _{ik}^M = \frac{{\delta _i^M}}{{1 + {\rm{e}}^{ \rho _k}}}} \end{array}$$

The counting process is also characterized as a drift–diffusion process, with a combination of individual and item-level effects. A min counting strategy is assumed to be used, where the counting is initiated from the larger addend to count up $n_k$ steps to the total, where $n_k$ is the smaller addend. For an item *k* the drift rate for counting is modeled as below, with $\delta _i^C$ representing the individuals counting efficiency:

4 $$\begin{array}{*{20}{c}} {\delta _{ik}^{C[{\rm{min}}]} = \frac{{\delta _i^C}}{{n_k}}} \end{array}$$

A selection of the counting strategy is accompanied by a larger non-decision time (relative to memory retrieval) to account for the strategy switching time. The strategy switching time is assumed to be a fixed time for each individual, with the assumption that memory retrieval is always attempted as a default strategy, but individuals may switch strategies at some point, and this switching time measures the persistence of individuals in sticking to a memory retrieval strategy (or lack of persistence and hence early switching away from memory retrieval). The decision threshold $\left( {\alpha _i} \right)$ is interpreted in terms of the degree of confidence required to decide and is assumed to be invariant to the choice of strategy used.

Since performance is measured at two time points with a relatively short time interval (8 weeks), the item *k* level parameters (obtained at the group level) are treated as objective difficulties that do not change over this time period. The basic non-decision time τ_*i*_ is also assumed to remain the same. However, the strategy switching non-decision time $\left( {\tau _i^C} \right)$, the decision threshold $\left( {\alpha _i} \right)$, as well as the individual strategy efficiencies $\left( {\delta _i^C,\;\delta _i^M} \right)$ and individual propensities for strategy use $\left( {\rho _i^M} \right)$ are allowed to vary between time points and individuals. Any effects of training and intervention are expected to be reflected in one or more of these five parameters. Response times less than 300 ms (less than 1% of the trials) were treated as missing values for the model inference. Using these parameters, the model posterior predictive accurately captured individual differences in reaction times and error rates (1 – accuracy) at both pre and post-training, showing the adequacy of model fit to data (Supplementary Fig. 5).

The joint brain behavioral model additionally builds in a latent regression of the change in brain modularity measure of interest against the change in all the model parameters, pre- and post-training (Supplementary Fig. 2).

Control analysis was performed to compare our model to two other models, a simple drift–diffusion model and a single strategy drift–diffusion model that accounts for variability in item-level difficulty but not the variability in the use of different strategies. This analysis revealed that our model accounting for both item difficulty and strategy dissociations provides the best fit to behavioral and brain data, compared to models that do not consider strategy-based dissociation of individual problems (Supplementary Table 4).

#### Model priors

Hierarchical normal priors, with appropriate truncation where required, were placed on memory propensity, memory efficacy, counting efficacy, and decision threshold for pre-training and for the change from pre- to post-training. Uniform priors were placed on non-decision time and switching time. Hierarchical normal priors were also placed on the item-level difficulty parameter. For the hierarchical priors, the hyperpriors used were uniform priors on the standard deviation and a multivariate normal prior for the hierarchical means. The multivariate normal hyperprior was constructed with normal priors on the means and an inverse Wishart prior on the covariance matrix of the multinormal. Markov chain Monte Carlo (MCMC) settings were 3 chains with 10,000 samples each, with a burn-in of 5000 (i.e., 5000 retained after burn-in) and a thinning factor of 1.

### Brain imaging

#### Functional MRI data acquisition

fMRI data were acquired using whole-brain imaging with a T2*-sensitive gradient echo spiral in/out pulse sequence at a Signa LX (GE Medical Systems) 3T scanner with the following parameters: echo time (TE) = 30 ms, repetition time (TR) = 2 s, flip angle = 80°, field-of-view = 200 mm, 29 axial-oblique slices parallel to the anterior commissure–posterior commissure line, dimensions 3.125 × 3.125 × 4 mm with 0.5-mm skip. To reduce blurring and signal loss from field inhomogeneity, an automated high order shimming method based on a spiral acquisition was used prior to the acquisition of functional MRI scans. Cushions were placed around participants’ heads to minimize head movement.

#### Structural MRI data acquisition

High-resolution T1-weighted images were acquired in each child at both scan sessions (that is, pre- and post-training) to facilitate anatomical co-registration of fMRI maps. A spoiled-gradient-recalled inversion recovery three-dimensional MRI sequence with the following parameters was used: *I* = 300 ms, TR = 8.4 ms; TE = 1.8 ms; flip angle = 15°; 22-cm field of view; 132 slices in coronal plane; 256 × 192 matrix; 2 NEX, acquired resolution = 1.5 × 0.9 × 1.1 mm.

#### Functional MRI task

The numerical problem-solving task was performed during fMRI. This task consisted of two runs of addition problem solving during which the child had to verify addition equations (for example, 3 + 4 = 7). Problems were presented in a fast event-related fMRI design with 12 single-digit addition problems per run. In each run, problems were presented horizontally in green lettering on a black background. In half of the problems, the answers presented were correct (for example, 2 + 4 = 6); in the remaining half, the answers presented deviated from the correct solution by ±1 or ±2 (for example, 3 + 5 = 7). Addition problems with 1 or 0 as operands were excluded. The larger operand was equally likely to appear in the first or second position. Each trial started with a fixation asterisk that lasted for 0.5 s. Then, the problem was presented for a maximum of 9.5 s, during which time the child could make the response. The participant used a response box to indicate if the answer was correct or not. After the response, the problem disappeared from the screen and a black screen appeared until the time window was filled to 9.5 s. A set of 12 problems constituting the Control condition was also presented during each run. These problems consisted of number identity verifications (for example, 7 = 7) and were randomly interspersed with the addition trials. Invalid trials were counterbalanced as in the Addition condition (that is, answers deviated from the correct solution by ±1 or ±2). This condition served as the control task for fMRI data analyses to better isolate brain activity related to numerical problem solving, controlling for low-level perceptual processing of visual stimuli and motor responses required to complete verification tasks. The task design also included a total of six rest periods—10 s each, which occurred at jittered intervals during each run to achieve an optimal event-related fMRI design. The rest periods were not explicitly modeled. Accuracy and median reaction times of correctly solved problems were computed separately for each participant for each of Addition and Control conditions (Supplementary Table 5). We used performance on Addition condition to assess numerical problem-solving ability.

#### Functional MRI preprocessing

Data were analyzed using SPM8 (http://www.fil.ion.ucl.ac.uk/spm/). The first five volumes were not analyzed to allow for signal equilibration. A linear shim correction was applied separately for each slice during reconstruction using a magnetic field map acquired automatically by the pulse sequence at the beginning of the scan. Images were realigned to correct for motion, corrected for errors in slice-timing, co-registered to each individual’s structural T1 images, spatially transformed to standard stereotaxic space (based on the Montreal Neurologic Institute coordinate system), resampled every 2 mm using sinc interpolation, and smoothed with a 6 mm full-width half-maximum Gaussian kernel to decrease spatial noise prior to statistical analysis. For co-registration, the individual’s highest quality-rated (that is, either before or after 8 weeks) structural MRI sequence was used.

Translational movement in millimeters (*x*,*y*,*z*), and rotational motion in degrees (pitch, roll, yaw) were calculated based on the SPM8 parameters for motion correction of the functional images of each subject. Mean scan-to-scan (framewise) displacement of movement did not exceed 1 mm for all participants in either session (that is, pre- or post-training) and was not significantly different between sessions (*t*(34) = 1.56, *p* = 0.31). Training-related change in head motion was not correlated with changes in latent and observable behavioral and brain measures (|*r*|s < .30, *p*s > 0.08; Supplementary Table 6). To correct for deviant volumes resulting from spikes in movement, we used de-spiking procedures similar to those implemented in AFNI^96^. Volumes with movement exceeding 0.5 voxels (1.562 mm) or spikes in global signal exceeding 5% were interpolated using adjacent scans.

#### Functional MRI network analysis: region of interest (ROI) selection

Neurosynth^77^-based meta-analysis using term “arithmetic” was used to identify 18 parietal, frontal, and ventral temporal-occipital regions consistently activated during numerical problem solving^1,13,32,40–43,47,97^, in addition to 12 hippocampus and parahippocampal gyral subregions defined in the Brainnetome atlas^98^ (Fig. 3a). Anatomical locations of the ROIs from the meta-analysis were identified by the Harvard-Oxford atlas.

#### Functional MRI network analysis: network construction

Psychophysiological interaction (PPI) connectivity analysis^99^ was performed using the 30 ROIs described above to construct a task-based numerical problem-solving brain network (Fig. 3a). We used a standard PPI analysis procedure^100–104^ which explicitly models and controls for overall task activation, and as such it models effective rather than synchronized task-related co-activation^99^. Specifically, our PPI analysis employed three regressors: a physiological variable representing the deconvolved time series within the seed region, a psychological variable representing *Addition* problem solving and *Control* number identity verification conditions, and a psychophysiological interaction term that represented the Hadamard cross-product of the first two regressors. PPI analyses were performed at the individual participant level and connectivity estimates corresponding to the *Addition* versus *Control* contrast were used as edge-weights of the 30 × 30 task-based functional connectivity of numerical problem-solving brain network.

#### Functional MRI network analysis: Graph-based analysis of global and regional modular organization

We used graph-theoretical and community detection techniques to investigate the global and regional measures of modular organization of functional connectivity among 30 node task-based numerical problem-solving brain network (Fig. 3a). Community detection was used to determine the optimal global modular structure within the functional connectivity matrix by grouping nodes into nonoverlapping communities or modules that maximize intramodular connectivity and minimize intermodular connectivity. The Louvain algorithm implemented in the Brain Connectivity Toolbox (http://www.brain-connectivity-toolbox.net) was used to detect community structure in the functional connectivity matrix. This algorithm optimizes a quality function Q*, defined as the difference between the observed intramodular connectivity and the intramodular connectivity expected by chance, while penalizing assignment of nodes with negative correlations to the same community. The Louvain algorithm automatically determines the number of underlying communities, and the resulting community structure is characterized by high positive and low negative connectivity within each community. It should be noted that this community structure was based on an unbiased weighted connectivity matrix, i.e., we did not impose an arbitrary threshold on the connectivity matrix. One commonly adopted and critical step in such analyses is to create a binary adjacency matrix by thresholding an association matrix (e.g., cross-correlation between brain nodes) at an arbitrary value. However, the use of such arbitrary thresholds is problematic, as it can lead to different levels of network sparsity and highly biased estimates of community structure. Our approach here overcomes these limitations.

Changes in large-scale modular network organization after 8 weeks were computed using an information-theoretic distance metric. Specifically, we computed the distance as one minus the mutual information between the modular organization at pre-training and the modular organization at post-training. Brain Connectivity Toolbox was used to compute the mutual information between two modular organizations.

Modular organization at the regional level was characterized by computing diversity coefficients of each of the 30 nodes belonging to the numerical problem-solving brain network. Diversity coefficient is a measure of how uniformly a brain region interacts with regions in other modules. Specifically, a high value for the diversity coefficient would indicate that interactions are more evenly distributed across modules. Diversity coefficient, is a more relevant measure than participation coefficient. Crucially, diversity coefficients are not influenced by the number of modules and thus consistent across different partitions of the same network^105^. In contrast, participation coefficients are influenced by the number of modules and thus variable across different partitions of the same network. Therefore, diversity coefficient is a more appropriate measure of regional modular connectivity in the current study which examines learning-induced changes in the partition of the brain network involved in numerical problem solving^105^. Brain Connectivity Toolbox was used to compute the diversity coefficient.

### Reporting summary

Further information on research design is available in the Nature Research Reporting Summary linked to this article.

## Supplementary information

Supplementary Information

Description of Additional Supplementary Files

Supplementary Data 1

Reporting Summary

## Data Availability

Source data for Figs. 1b, 2b, 4, 5a–d, and 6a–d have been provided in Supplementary Data 1. All the other data that support the findings of this study are available from the corresponding authors upon reasonable request.

## Code availability

Data were analyzed using Matlab 9.5 (R2018b), JAGS 4.3.0, SPM8, and Brain Connectivity Toolbox. Data analysis scripts are available from the corresponding authors upon reasonable request.
