# Supplementary material for: Neurocognitive modeling of latent memory processes reveals reorganization of hippocampal-cortical circuits underlying learning and efficient strategies
Source: Commun Biol. 2021 Mar 25;4:405. doi: 10.1038/s42003-021-01872-1 (PMC7994581; doi:10.1038/s42003-021-01872-1)
Supplement: Supplementary file 1 — Supplementary Information [file 42003_2021_1872_MOESM1_ESM.pdf]

# Supplementary Information

# Neurocognitive modeling of latent memory processes reveals reorganization of hippocampal-cortical circuits underlying learning and efficient strategies

Kaustubh Supekar[1Ψ*], Hyesang Chang[1Ψ], Percy K Mistry[1Ψ], Teresa Iuculano[1,2], Vinod Menon[1,3*]

[1] Department of Psychiatry & Behavioral Sciences, Stanford University, Stanford, California, United States of America

[2] Developmental Psychology and Child Education Laboratory, University Paris Descartes, Paris, France

[3] Stanford Neuroscience Institute, Stanford University, Stanford, California, United States of America

[*] Corresponding authors

E-mail: ksupekar@stanford.edu (KS), menon@stanford.edu (VM)

[Ψ] These authors contributed equally to this work

# Supplementary Figures

**Supplementary Figure 1. Bayesian graphical schematic for cognitive process model.**

;

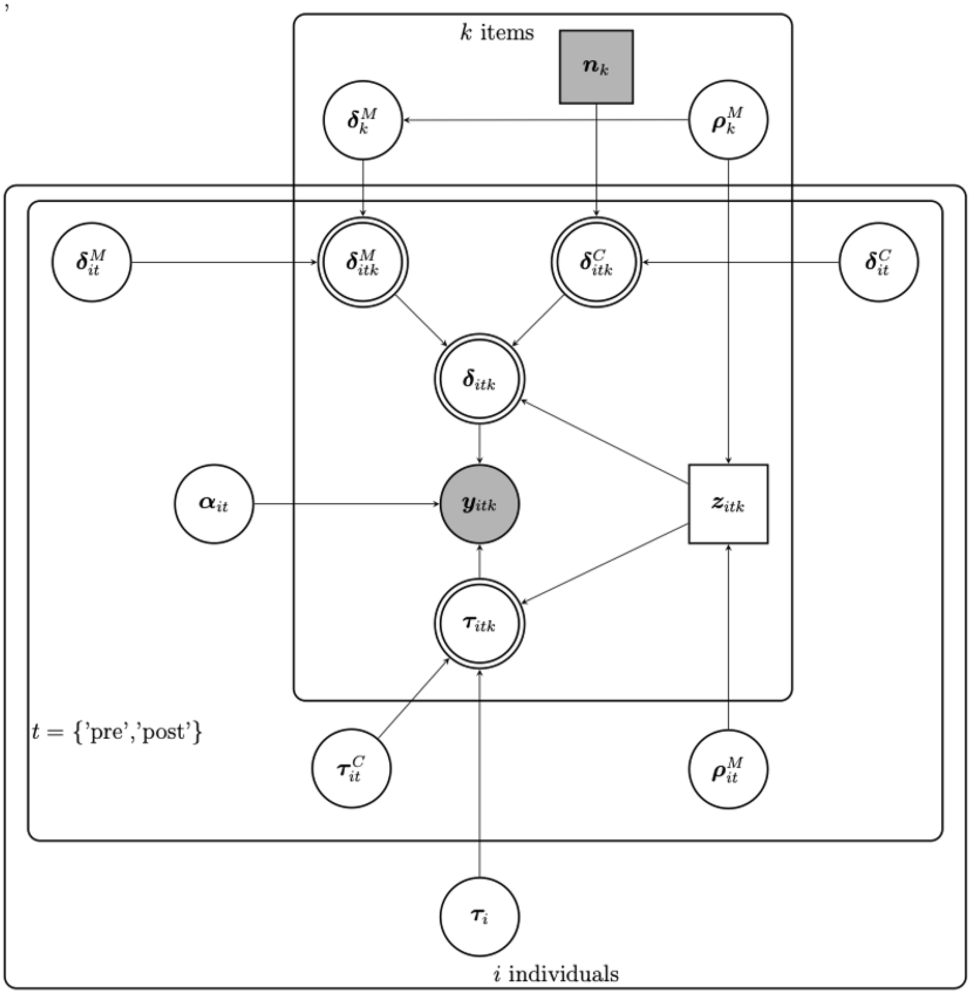


*Abbreviations*:

$\delta_{it}^M$ = Individual component of memory retrieval efficiency, independent of item difficulty

$\delta_{it}^C$ = Individual component of counting efficiency, independent of item difficulty

$\alpha_{it}$ = Decision threshold for an individual

$\tau_{it}^C$ = Strategy switching time (additional time taken to switch from memory to counting strategy)

$\tau_i$ = Non-decision time

$\rho_{it}^M$ = Individual component of memory retrieval propensity, independent of item difficulty

$\rho_k^M$ = Item-level memory retrieval difficulty

$z_{itk}$ = Selection of strategy on each trial (memory vs counting) based on $p(\text{memory}) = \frac{1}{1+e^{-(\rho_i - \rho_k)}}$

$n_k$ = Lower addend

A graphical illustration of the cognitive model of the addition problem solving process, where $y_{itk}$ represents the observed choice (accuracy) coded response times for the $i^{\text{th}}$ individual for item *k,* with the index *t* representing pre- versus post-training performance. The choice coded response time provides a joint distribution of accuracy and response times, and is modeled using a drift diffusion model (DDM), with the key parameters drift rate ($\delta$), decision threshold ($\alpha$), and non-decision time ($\tau$). The actual drift rate and non-decision time applicable on any particular trial are dependent on the inferred strategy used, denoted by the discrete node (*z*). For memory retrieval, the drift process is governed by an individuals' memory retrieval efficiency ($\delta_{it}^{M}$) and the item level retrieval difficulty ($\rho_k^M$) inferred at a group level. For counting, the drift process is governed by an individuals' counting efficiency ($\delta_{it}^{C}$), the observed lower addend ($n_k$) assuming a min-counting strategy (that is, counting up from the higher addend), and the strategy switching time from autonomous retrieval to counting ($\tau_{it}^{C}$). The decision threshold ($\alpha_{it}$) and non-decision time ($\tau_i$) are common to both processes. The memory propensity ($\rho_{it}^{M}$) and item difficulty ($\rho_k^M$) parameter together govern the individuals' probability of using memory versus counting on any particular trial. The item difficulty parameter ($\rho_k^M$) is a common cause, affecting both the probability and efficiency of memory retrieval.

**Supplementary Figure 2. Bayesian graphical schematic for joint neurocognitive process model.**

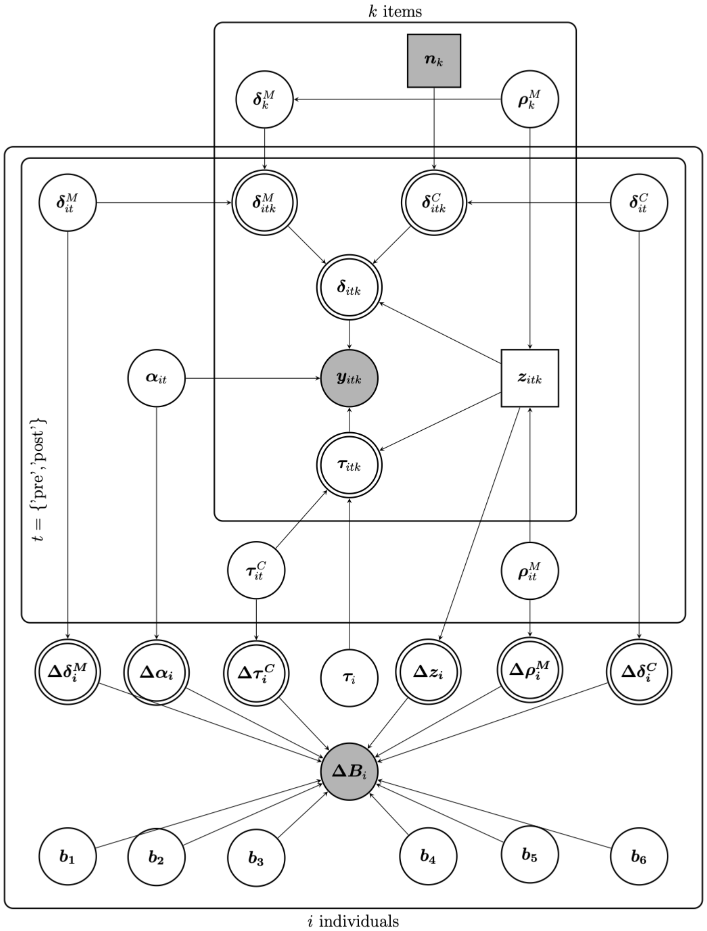


A graphical model of the cognitive model of the addition process, augmented with a statistical link between the change in cognitive parameters and change in brain features. The coefficients $b_1$ to $b_6$ are inferred jointly along with the cognitive model parameters within a Bayesian inference framework, and measure the multivariate relationship between changes in the cognitive parameters and how well these changes can explain the changes in the hippocampal connectivity between pre- and post- training ($\Delta B_i$). Abbreviations are the same as in Supplementary Figure 1.

**Supplementary Figure 3. Cognitive training related changes in network organization of all nodes.**

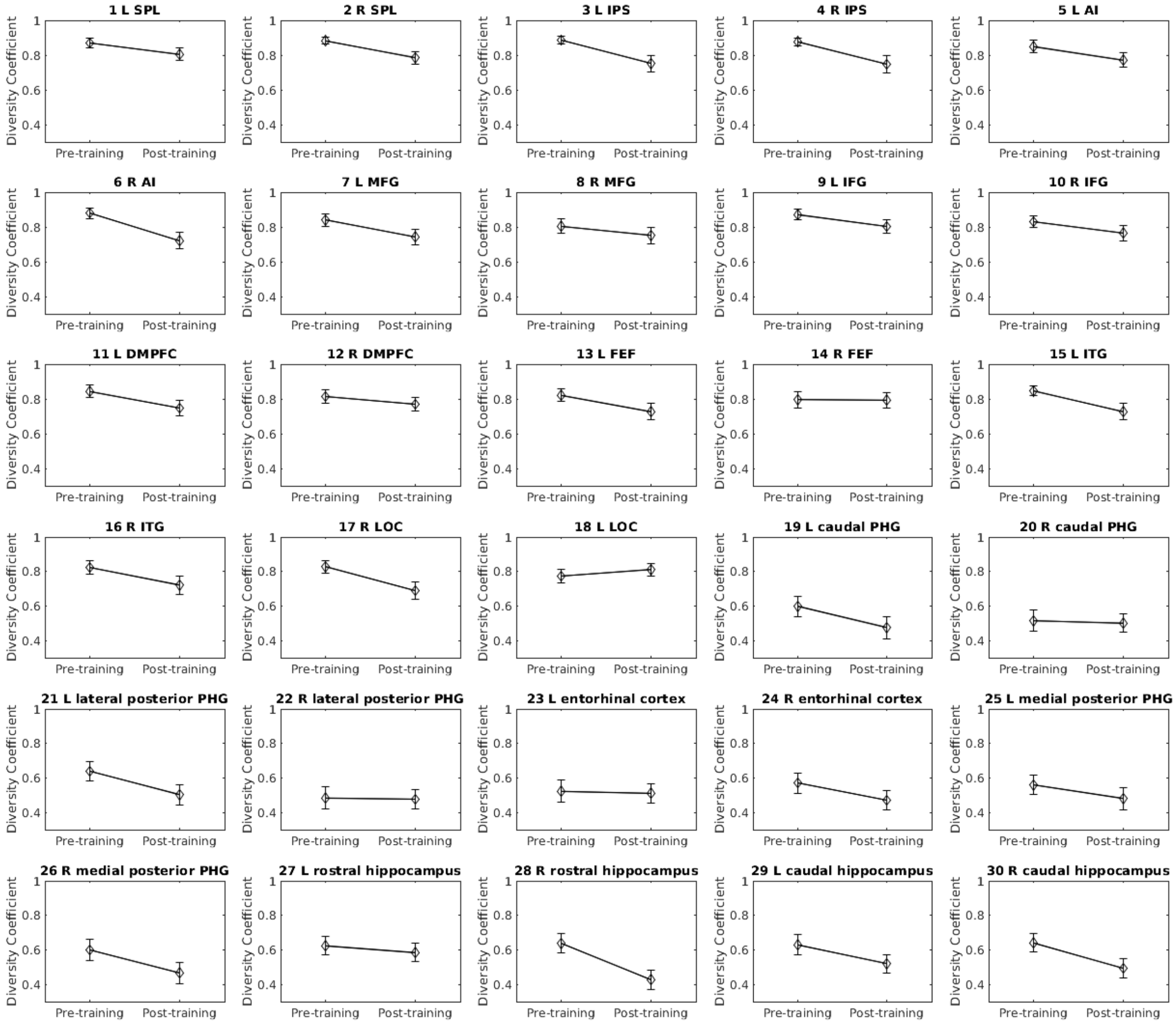


Error bar shows standard error of mean.

**Supplementary Figure 4. Characterization of changes in brain network modular organization based on canonical drift diffusion model (DDM) and item- and strategy-dissociation model.**

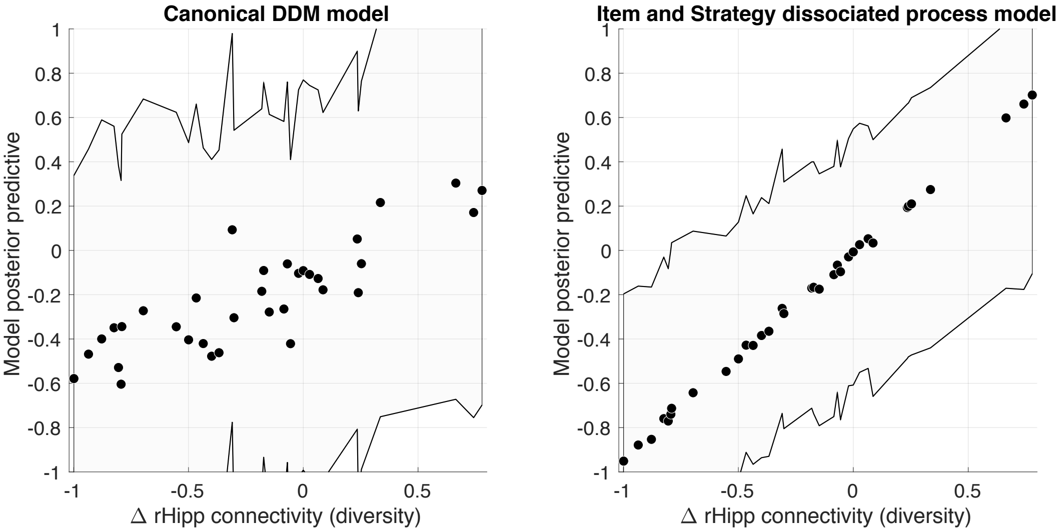


The posterior predictive value for changes in the hippocampal connectivity (diversity coefficient) between pre- and post- training ($\Delta B_i$) as generated by the model based on inferred model parameters (i.e. the posterior distribution of the changes in hippocampal connectivity generated by the model, *y-axis*), against the actual changes (*x-axis*). The posterior distribution depends on the inferred coefficients ($b_1$ to $b_6$, Supplementary Figure 2), and the changes in cognitive parameters inferred from behavioral data. The figure on the left shows the results from a control model where no strategy selection was implemented (i.e. a simple single strategy DDM), and the figure on the right shows the results of the model shown in Supplementary Figure 2, which implements differentiation between memory and counting strategies. Allowing for strategy differences within the cognitive model allows us to capture a far more precise basis for the changes in hippocampal connectivity observed post training.

**Supplementary Figure 5. Model posterior predictive for reaction times and error rates**

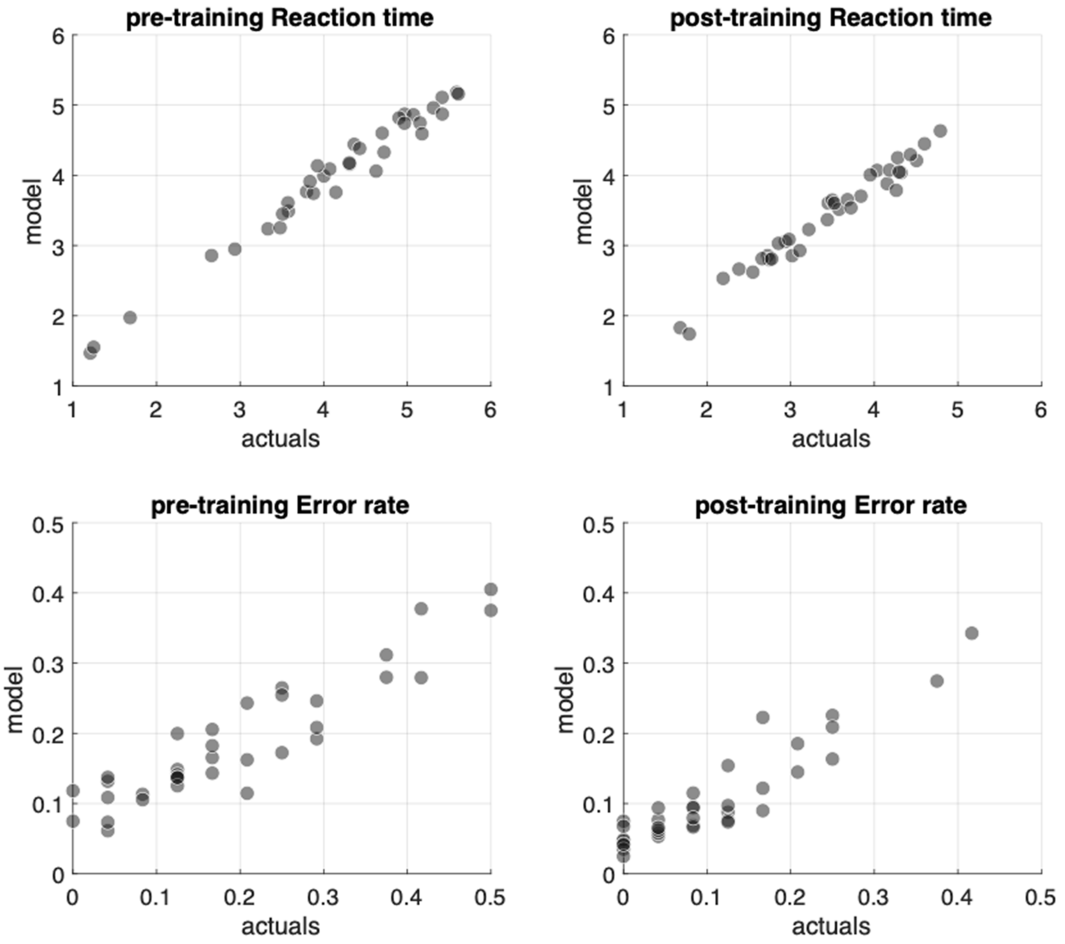


Model posterior predictive accurately capture individual differences in reaction times and error rates (1 – accuracy) at both pre and post training, showing the adequacy of model fit to data.

# Supplementary Tables

**Supplementary Table 1. Correlations between training-related change in latent cognitive measures (memory retrieval strategy use and efficiency) and training-related change in observable behavioral measures: accuracy and reaction time.**

| | Δ Memory Retrieval Strategy Use | Δ Memory Retrieval Efficiency |
|---|---|---|
| Δ Accuracy | ρ = -0.11, $p$ = 0.51 | ρ = 0.42, $p$ = 0.01 |
| Δ Reaction time | ρ = -0.49, $p$ = 0.01 | ρ = 0.08, $p$ = 0.64 |

**Supplementary Table 2. Correlations between neuropsychological measures and training-related changes in latent and observable behavioral and brain measures.**

| | Full-scale IQ | Verbal IQ | Performance IQ | Word reading | Numerical Ops | Math Reasoning |
|---|---|---|---|---|---|---|
| Changes in observable behavioral measures | | | | | | |
| Δ Accuracy | $\rho = -0.13$<br>$p = 0.46$ | $\rho = 0.11$<br>$p = 0.52$ | $\rho = -0.24$<br>$p = 0.15$ | $\rho = -0.27$<br>$p = 0.12$ | $\rho = -0.18$<br>$p = 0.31$ | $\rho = -0.30$<br>$p = 0.07$ |
| Δ RT | $\rho = 0.22$<br>$p = 0.19$ | $\rho = 0.19$<br>$p = 0.28$ | $\rho = 0.13$<br>$p = 0.43$ | $\rho = 0.05$<br>$p = 0.77$ | $\rho = -0.08$<br>$p = 0.63$ | $\rho = -0.07$<br>$p = 0.67$ |
| Changes in latent behavioral measures | | | | | | |
| Δ Memory Retrieval Efficiency | $\rho = 0.11$<br>$p = 0.51$ | $\rho = 0.05$<br>$p = 0.76$ | $\rho = 0.08$<br>$p = 0.65$ | $\rho = 0.04$<br>$p = 0.79$ | $\rho = 0.18$<br>$p = 0.30$ | $\rho = 0.03$<br>$p = 0.83$ |
| Δ Memory Retrieval Strategy Use | $\rho = 0.07$<br>$p = 0.71$ | $\rho = -0.03$<br>$p = 0.86$ | $\rho = 0.18$<br>$p = 0.31$ | $\rho = -0.02$<br>$p = 0.95$ | $\rho = 0.09$<br>$p = 0.63$ | $\rho = 0.07$<br>$p = 0.70$ |
| Changes in brain measures | | | | | | |
| Δ Modular Brain Network Organization | $\rho = -0.06$<br>$p = 0.72$ | $\rho = 0.20$<br>$p = 0.26$ | $\rho = -0.23$<br>$p = 0.18$ | $\rho = -0.34$<br>$p = 0.05$ | $\rho = -0.09$<br>$p = 0.60$ | $\rho = -0.28$<br>$p = 0.11$ |
| Δ Hippocampus Network Organization | $\rho = -0.19$<br>$p = 0.27$ | $\rho = -0.14$<br>$p = 0.41$ | $\rho = -0.16$<br>$p = 0.33$ | $\rho = -0.01$<br>$p = 0.95$ | $\rho = -0.04$<br>$p = 0.78$ | $\rho = 0.12$<br>$p = 0.47$ |

**Supplementary Table 3. Demographic and cognitive profile.**

| | Mean (± standard error of the mean) |
|---|---|
| Age | 8.58 (±0.09) |
| Gender (Males/Females) | 15/20 |
| Grade | 3.00 (±0.00) |
| | |
| WASI | |
| Full scale IQ | 106.3 (±2.19) |
| | |
| WIAT-II | |
| Word Reading | 105.2 (±1.73) |
| Reading Comprehension | 106.7 (±1.76) |
| Numerical Operations | 95.9 (±2.62) |
| Math Reasoning | 101.5 (±2.28) |

Abbreviations: WASI = Wechsler Abbreviated Scale of Intelligence ($1^{st}$ edition); WIAT-II = Wechsler Individual Achievement Test ($2^{nd}$ edition).

**Supplementary Table 4. Model Comparison: Item and strategy dissociation-based drift diffusion model (DDM) provides the best description of behavioral (change in accuracy) and brain (change in right rostral hippocampal diversity coefficient) data based on the deviance information criterion (DIC) which considers both model fit and complexity, compared to models that do not consider strategy-based dissociation of individual problems.**

| | Canonical DDM | Item-based DDM | Item- and strategy-based DDM |
|---|---|---|---|
| DIC Behavioral (lower is better) | 6890 | 6752 | 6055 |
| DIC Behavioral + Brain (lower is better) | 6949 | 6781 | 6080 |

**Supplementary Table 5. Performance on fMRI task (addition and control conditions).**

| | Pre-training | Post-training |
|---|---|---|
| Addition condition: Accuracy | 0.81 (±0.02) | 0.90 (±0.02) |
| Addition condition: Reaction time (ms) | 3835 (±204) | 3104 (±142) |
| Control condition: Accuracy | 0.93 (±0.01) | 0.94 (±0.01) |
| Control condition: Reaction time (ms) | 1927 (±94) | 1627 (±75) |

Mean (±Standard error of the mean)

**Supplementary Table 6. Relationship between training-related change in mean framewise displacement and training-related change in latent and observable behavioral and brain measures.**

| | Δ Framewise displacement |
|---|---|
| Δ Accuracy | $r = -0.24$, $p = 0.16$ |
| Δ Reaction time | $r = -0.22$, $p = 0.20$ |
| Δ Memory retrieval efficiency | $r = -0.29$, $p = 0.09$ |
| Δ Memory retrieval strategy use | $r = 0.26$, $p = 0.13$ |
| Δ Modular brain network organization | $r = -0.17$, $p = 0.34$ |
| Δ Hippocampus network organization | $r = 0.05$, $p = 0.78$ |

## Supplementary Discussion

### ***Theoretically motivated constraints on inference about the use of latent strategies***

Our computational methods for trial-by-trial dissociation of latent strategy use allow us to measure the relationships between different strategy specific process components, and both improvements in behavior and changes in brain connectivity, post training. This process dissociation model relies on imposing theoretically-motivated constraints on inference about which latent strategy is being used on a trial-by-trial basis. Each strategy (memory and counting) is associated with different drift rates and non-decision times (Figure 2a; Supplementary Figures 1-2). The memory strategy is assumed to be the autonomous or default strategy. An individual may switch to a counting strategy after a minimum strategy switching time, that is a model-inferred individual level parameter, and remains the same for an individual across all items. Thus, the non-decision time for a counting strategy is equal to the non-decision time for memory retrieval plus the strategy switching time. The drift rates for both memory and counting are different, and each is composed of both an individual component and an item component. This allows us to frame the drift rates within an item-response structure. The individual components for memory and counting reflect an individuals' ability for each strategy, controlling for differences in item difficulty, and is inferred separately before and after training. The item component remains the same for all participants, and remains the same before and after training. It is designed to reflect the objective item level difficulties independent of individual ability, but reflecting a population level indicator. Further, the item difficulties for counting are simply a linear function of the lower addend, that is, across all items, the counting drift rate for all individuals falls in a manner inversely proportional to the lower addend. For memory retrieval, the item difficulties are inferred by the model. The easier an item, the higher the memory drift

rate, with a logistic relationship between memory difficulty and memory drift rate. The model assumes a common cause structure, such that the memory difficulty parameter affects not just the memory drift rate, but also the probability of memory use. This probability is based on a logistic relationship with the difference between an individual propensity for using memory and the memory linked item difficulty. This probability is assumed to define which strategy is selected by an individual on a particular trial. For model inference, this forms a unique prior on the strategy use for each individual and item combination, and the posterior probability of strategy use is inferred based on this probability, and whether the individual components of drift, item level difficulties, the individual component of memory propensity, and strategy switching time for the memory versus counting strategies better explain the accuracy and reaction time behavior on each trial. Successfully applying this model requires each individual to complete a reasonable number of unique items (so that individual components across items can be robustly estimated) and that each unique item is completed by a reasonable number of individuals (so that item components across individuals can be robustly estimated). Under these conditions, the model can infer which strategy use (and associated strategy-level parameters) is more probable given the observed data.

The computational modeling approach we developed here thus overcomes key limitations of previous methods and addresses critical challenges thereby providing a quantitative template for investigating latent cognitive processes in other domains.
