# Supplementary material for: Neurocognitive modeling of latent memory processes reveals reorganization of hippocampal-cortical circuits underlying learning and efficient strategies
Source: Commun Biol. 2021 Mar 25;4:405. doi: 10.1038/s42003-021-01872-1 (PMC7994581; doi:10.1038/s42003-021-01872-1)
Supplement: Supplementary file 2 — Description of Additional Supplementary Files [file 42003_2021_1872_MOESM2_ESM.pdf]

Description of Additional Supplementary Files

File name: Supplementary Data 1

Description: Source data for Figs 1b, 2b, 4, 5a-d, and 6a-d.
