# Supplementary material for: Neurocognitive modeling of latent memory processes reveals reorganization of hippocampal-cortical circuits underlying learning and efficient strategies
Source: Commun Biol. 2021 Mar 25;4:405. doi: 10.1038/s42003-021-01872-1 (PMC7994581; doi:10.1038/s42003-021-01872-1)
Supplement: Supplementary file 4 — Reporting Summary [file 42003_2021_1872_MOESM4_ESM.pdf]

nature research

Corresponding author(s): Kaustubh Supekar, Vinod Menon

Last updated by author(s): Jan 27, 2021

Reporting Summary

Nature Research wishes to improve the reproducibility of the work that we publish. This form provides structure for consistency and transparency in reporting. For further information on Nature Research policies, see our Editorial Policies and the Editorial Policy Checklist.

Statistics

For all statistical analyses, confirm that the following items are present in the figure legend, table legend, main text, or Methods section.

n/a	Confirmed	
☐	☒	The exact sample size (n) for each experimental group/condition, given as a discrete number and unit of measurement
☐	☒	A statement on whether measurements were taken from distinct samples or whether the same sample was measured repeatedly
☐	☒	The statistical test(s) used AND whether they are one- or two-sided<br>*Only common tests should be described solely by name; describe more complex techniques in the Methods section.*
☐	☒	A description of all covariates tested
☐	☒	A description of any assumptions or corrections, such as tests of normality and adjustment for multiple comparisons
☐	☒	A full description of the statistical parameters including central tendency (e.g. means) or other basic estimates (e.g. regression coefficient) AND variation (e.g. standard deviation) or associated estimates of uncertainty (e.g. confidence intervals)
☐	☒	For null hypothesis testing, the test statistic (e.g. F, t, r) with confidence intervals, effect sizes, degrees of freedom and P value noted<br>*Give P values as exact values whenever suitable.*
☐	☒	For Bayesian analysis, information on the choice of priors and Markov chain Monte Carlo settings
☒	☐	For hierarchical and complex designs, identification of the appropriate level for tests and full reporting of outcomes
☐	☒	Estimates of effect sizes (e.g. Cohen's d, Pearson's r), indicating how they were calculated

Our web collection on statistics for biologists contains articles on many of the points above.

Software and code

Policy information about availability of computer code

Data collection	Behavioral data were collected using standardized assessments including the Wechsler Abbreviated Scale of Intelligence (WASI, 1st edition) and the Wechsler Individual Achievement Test (WIAT, 2nd edition). Brain imaging data were acquired on a 3T GE scanner. Numerical problem solving task was presented in the fMRI scanner.
Data analysis	Behavioral data were analyzed using Matlab and JAGS. Brain imaging data were preprocessed using SPM8 and analyzed using Matlab and the Brain Connectivity Toolbox.

For manuscripts utilizing custom algorithms or software that are central to the research but not yet described in published literature, software must be made available to editors and reviewers. We strongly encourage code deposition in a community repository (e.g. GitHub). See the Nature Research guidelines for submitting code & software for further information.

Data

Policy information about availability of data

All manuscripts must include a data availability statement. This statement should provide the following information, where applicable:

- Accession codes, unique identifiers, or web links for publicly available datasets
- A list of figures that have associated raw data
- A description of any restrictions on data availability

Source data for Figs 1b, 2b, 4, 5a-d, and 6a-d have been provided in Supplementary Data 1. All the other data that support the findings of this study are available from the corresponding authors upon reasonable request.

Field-specific reporting

Please select the one below that is the best fit for your research. If you are not sure, read the appropriate sections before making your selection.

☒ Life sciences ☐ Behavioural & social sciences ☐ Ecological, evolutionary & environmental sciences

For a reference copy of the document with all sections, see nature.com/documents/nr-reporting-summary-flat.pdf

Life sciences study design

All studies must disclose on these points even when the disclosure is negative.

Sample size	The sample size (35 children) were determined based on samples from previous neuroimaging-based cognitive training studies in children. Based on prior research with effect size of Cohen's d = 1.2 (Iuculano et al., 2015), the estimated power to detect significant associations between intervention-induced brain plasticity associated with learning with 35 children is 92%.
Data exclusions	The exclusion criteria were established in previous studies. No data were additionally excluded in the current study.<br><br>In previous studies, the following participants were excluded:<br>Left-handed participants.<br>Participants with history of psychiatric illness, neurological disorders or reading disabilities.<br>Participants with incomplete/missing behavioral or imaging data.<br>Participants with poor behavioral performance, poor quality imaging data or excessive head movement.
Replication	The current study did not test reproducibility of the findings.
Randomization	A one group pretest-posttest design was employed in the current study.
Blinding	Blinding is not relevant to the design of the current study.

Reporting for specific materials, systems and methods

We require information from authors about some types of materials, experimental systems and methods used in many studies. Here, indicate whether each material, system or method listed is relevant to your study. If you are not sure if a list item applies to your research, read the appropriate section before selecting a response.

Materials & experimental systems

n/a	Involved in the study	
☒	☐	Antibodies
☒	☐	Eukaryotic cell lines
☒	☐	Palaeontology and archaeology
☒	☐	Animals and other organisms
☐	☒	Human research participants
☒	☐	Clinical data
☒	☐	Dual use research of concern

Methods

n/a	Involved in the study	
☒	☐	ChIP-seq
☒	☐	Flow cytometry
☐	☒	MRI-based neuroimaging

Human research participants

Policy information about studies involving human research participants

Population characteristics	Thirty-five children in grade 3 (mean age 8.58, 20 females). Participants had no history of psychiatric illness, neurological disorders or reading disabilities.
Recruitment	Participants were recruited from multiple school districts in the San Francisco Bay Area.
Ethics oversight	Informed consent was obtained from the legal guardian of each child and all study protocols were approved by the Stanford University Institutional Review Board.

Note that full information on the approval of the study protocol must also be provided in the manuscript.

Magnetic resonance imaging

Experimental design

Design type	Event-related task fMRI
Design specifications	The numerical problem-solving task was performed during fMRI. This task consisted of two runs of addition problem solving during which the child had to verify addition equations (for example, 3+4=7). Problems were presented in a fast event-related fMRI design with 12 single-digit addition problems per run. In each run, problems were presented horizontally in green lettering on a black background. In half of the problems, the answers presented were correct (for example, 2+4=6); in the remaining half, the answers presented deviated from the correct solution by ±1 or ±2 (for example, 3+5=7). Addition problems with 1 or 0 as operands were excluded. The larger operand was equally likely to appear in the first or second position. Each trial started with a fixation asterisk that lasted for 0.5 s. Then, the problem was presented for a maximum of 9.5 s, during which time the child could make the response. The participant used a response box to indicate if the answer was correct or not. After the response, the problem disappeared from the screen and a black screen appeared until the time window was filled to 9.5 s. A set of 12 problems constituting the Control condition was also presented during each run. These problems consisted of number identity verifications (for example, 7=7) and were randomly interspersed with the addition trials. Invalid trials were counterbalanced as in the Addition condition (that is, answers deviated from the correct solution by ±1 or ±2). This condition served as the control task for fMRI data analyses to better isolate brain activity related to numerical problem solving, controlling for low-level perceptual processing of visual stimuli and motor responses required to complete verification tasks. The task design also included a total of six rest periods—10 s each —, which occurred at jittered intervals during each run to achieve an optimal event-related fMRI design.
Behavioral performance measures	Accuracy and median reaction times of correctly solved problems were computed separately for each participant for each of Addition and Control conditions. We used performance on Addition condition to assess numerical problem-solving ability.

Acquisition

Imaging type(s)	Functional; Structural
Field strength	3T
Sequence & imaging parameters	Functional: T2*-sensitive gradient echo spiral in/out pulse sequence, echo time (TE)=30 ms, repetition time (TR)=2 s, flip angle=80°, field-of-view=200 mm, 29 axial-oblique slices parallel to the AC–PC, dimensions 3.125 × 3.125 × 4 mm with 0.5-mm skip.<br><br>Structural: spoiled-gradient-recalled inversion recovery three-dimensional (3D) MRI sequence, I=300 ms, TR=8.4 ms; TE=1.8 ms; flip angle=15°; 22-cm field of view; 132 slices in coronal plane; 256 × 192 matrix; 2 NEX, acquired resolution=1.5 × 0.9 × 1.1 mm.
Area of acquisition	Whole brain scan

Diffusion MRI ☐ Used ☒ Not used

Preprocessing

Preprocessing software	Data were analyzed using SPM8 (http://www.fil.ion.ucl.ac.uk/spm/). The first five volumes were not analyzed to allow for signal equilibration. A linear shim correction was applied separately for each slice during reconstruction using a magnetic field map acquired automatically by the pulse sequence at the beginning of the scan. Images were realigned to correct for motion, corrected for errors in slice-timing, co-registered to each individual's structural T1 images, spatially transformed to standard stereotaxic space (based on the Montreal Neurologic Institute coordinate system), resampled every 2 mm using sinc interpolation, and smoothed with a 6 mm full-width half-maximum Gaussian kernel to decrease spatial noise prior to statistical analysis.
Normalization	Data were normalized to standard stereotaxic space
Normalization template	MNI
Noise and artifact removal	Images were smoothed with a 6 mm full-width half-maximum Gaussian kernel to decrease spatial noise.
Volume censoring	To correct for deviant volumes resulting from spikes in movement, we used de-spiking procedures similar to those implemented in AFNI. Volumes with movement exceeding 0.5 voxels (1.562 mm) or spikes in global signal exceeding 5% were interpolated using adjacent scans.

Statistical modeling & inference

Model type and settings	A standard PPI connectivity analysis was performed as described in 'Functional and/or Effective connectivity' section.

Effect(s) tested

Connectivity estimates corresponding to the Addition versus Control contrast were used as edge-weights of the 30 x 30 task-based functional connectivity of numerical problem-solving brain network.

Specify type of analysis: ☐ Whole brain ☒ ROI-based ☐ Both

Anatomical location(s)

Anatomical locations of regions of interest from Neurosynth-based meta-analysis were identified by the Harvard-Oxford atlas. The medial temporal lobe subdivisions were defined in the Brainnetome atlas.

Statistic type for inference
(See Eklund et al. 2016)

Graph-theoretical and community detection techniques were used to determine the modular organization of the numerical problem-solving brain network constructed from PPI analysis as described in 'Graph analysis' section.

Correction

False discovery rate (FDR) correction was applied across the ROIs tested.

Models & analysis

n/a	Involved in the study
☐	☒ Functional and/or effective connectivity
☐	☒ Graph analysis
☒	☐ Multivariate modeling or predictive analysis

Functional and/or effective connectivity

We used a standard PPI analysis procedure which explicitly models and controls for overall task activation, and as such it models effective rather than synchronized task-related co-activation. Specifically, our PPI analysis employed three regressors: a physiological variable representing the deconvolved time series within the seed region, a psychological variable representing Addition problem solving and Control number identity verification conditions, and a psychophysiological interaction term that represented the Hadamard cross-product of the first two regressors. Thirty pre-defined regions of interest were used to construct a task-based numerical problem-solving brain network PPI analyses were performed at the individual participant level and connectivity estimates corresponding to the Addition versus Control contrast were used as edge-weights of the 30 x 30 task-based functional connectivity of numerical problem-solving brain network.

Graph analysis

We used graph-theoretical and community detection techniques to investigate the global and regional measures of modular organization of functional connectivity among 30 node task-based numerical problem-solving brain network.

Community detection was used to determine the optimal global modular structure within the functional connectivity matrix by grouping nodes into nonoverlapping communities or modules that maximize intramodular connectivity and minimize intermodular connectivity. The Louvain algorithm implemented in the Brain Connectivity Toolbox was used to detect community structure in both the static and time-varying connectivity matrices. This community structure was based on an unbiased weighted connectivity matrix, i.e., we did not impose an arbitrary threshold on the connectivity matrix.

Changes in large-scale modular network organization after 8 weeks were computed using an information-theoretic distance metric. Specifically, we computed the distance as one minus the mutual information between the modular organization at pre-training and the modular organization at post-training. Brain Connectivity Toolbox was used to compute the mutual information between two modular organizations.

Modular organization at the regional level was characterized by computing diversity coefficients of each of the 30 nodes belonging to the numerical problem-solving brain network. Diversity coefficient is a measure of how uniformly a brain region interacts with regions in other modules. Specifically, a high value for the diversity coefficient would indicate that interactions are more evenly distributed across modules. Brain Connectivity Toolbox was used to compute the diversity coefficient.
